# Diagnostic Performance of Electronic Noses in Cancer Diagnoses Using Exhaled Breath: A Systematic Review and Meta-analysis

**DOI:** 10.1001/jamanetworkopen.2022.19372

**Published:** 2022-06-29

**Authors:** Max H. M. C. Scheepers, Zaid Al-Difaie, Lloyd Brandts, Andrea Peeters, Bart van Grinsven, Nicole D. Bouvy

**Affiliations:** 1 GROW School for Oncology and Developmental Biology, Maastricht University, Maastricht, the Netherlands; 2 Department of Clinical Epidemiology and Medical Technology Assessment, Maastricht University Medical Centre, the Netherlands; 3 Sensor Engineering, Faculty of Science and Engineering, Maastricht University, Maastricht, the Netherlands; 4 Department of Surgery, Maastricht University Medical Center, Maastricht, the Netherlands

## Abstract

**Importance:**

There has been a growing interest in the use of electronic noses (e-noses) in detecting volatile organic compounds in exhaled breath for the diagnosis of cancer. However, no systematic evaluation has been performed of the overall diagnostic accuracy and methodologic challenges of using e-noses for cancer detection in exhaled breath.

**Objective:**

To provide an overview of the diagnostic accuracy and methodologic challenges of using e-noses for the detection of cancer.

**Data Sources:**

An electronic search was performed in the PubMed and Embase databases (January 1, 2000, to July 1, 2021).

**Study Selection:**

Inclusion criteria were the following: (1) use of e-nose technology, (2) detection of cancer, and (3) analysis of exhaled breath. Exclusion criteria were (1) studies published before 2000; (2) studies not performed in humans; (3) studies not performed in adults; (4) studies that only analyzed biofluids; and (5) studies that exclusively used gas chromatography–mass spectrometry to analyze exhaled breath samples.

**Data Extraction and Synthesis:**

PRISMA guidelines were used for the identification, screening, eligibility, and selection process. Quality assessment was performed using Quality Assessment of Diagnostic Accuracy Studies 2. Generalized mixed-effects bivariate meta-analysis was performed.

**Main Outcomes and Measures:**

Main outcomes were sensitivity, specificity, and mean area under the receiver operating characteristic curve.

**Results:**

This review identified 52 articles with a total of 3677 patients with cancer. All studies were feasibility studies. The sensitivity of e-noses ranged from 48.3% to 95.8% and the specificity from 10.0% to 100.0%. Pooled analysis resulted in a mean (SE) area under the receiver operating characteristic curve of 94% (95% CI, 92%-96%), a sensitivity of 90% (95% CI, 88%-92%), and a specificity of 87% (95% CI, 81%-92%). Considerable heterogeneity existed among the studies because of differences in the selection of patients, endogenous and exogenous factors, and collection of exhaled breath.

**Conclusions and Relevance:**

Results of this review indicate that e-noses have a high diagnostic accuracy for the detection of cancer in exhaled breath. However, most studies were feasibility studies with small sample sizes, a lack of standardization, and a high risk of bias. The lack of standardization and reproducibility of e-nose research should be addressed in future research.

## Key Points

**Question:**

What are the diagnostic performances and methodologic challenges of using electronic noses (e-noses) to detect cancer in exhaled breath?

**Findings:**

This systematic review and meta-analysis identified 52 studies with 3677 patients with cancer, from which pooled analysis found e-noses to have a sensitivity of 90% and a specificity of 87% in detecting cancer. Most studies were feasibility studies with small sample sizes and poor standardization.

**Meaning:**

Although e-noses were found to have promising accuracy in detecting cancer, there is a need for standardized external validation studies that evaluate their diagnostic accuracy so that their role in the diagnostic workup of cancer can be established.

## Teaser

This systematic review and meta-analysis evaluates the diagnostic performance of electronic nose technologies used for cancer diagnosis in exhaled breath.

## Introduction

Interest in the use of volatile organic compounds (VOCs) in exhaled breath to diagnose cancer has been increasing.^1,2^ Volatile organic compounds are degradation products of biochemical processes in the human body that can be detected in exhaled breath. The composition of VOCs in exhaled breath changes because of pathological processes, such as cancer.^3^ Because of their low solubility in blood, VOCs diffuse easily into alveolar air and are subsequently excreted via exhaled breath, enabling detection.^4^ Disease-specific differences in VOC profiles enable scientists to investigate breath for the detection of specific conditions, including cancer.

Breath analysis has several advantages compared with analysis of other clinical samples. Breath testing is painless and noninvasive. In addition, unlike blood and urine samples, breath samples do not need any workup, allowing for immediate analysis and rapid results.

The most accurate method for identifying VOCs is a combination of gas chromatography–mass spectrometry (GC-MS),^5^ which is highly accurate and allows for the selective detection of individual VOCs. Previous studies^6,7,8,9^ that used GC-MS have identified potentially disease-specific VOCs as indicators of several malignant tumors. However, GC-MS is both time-consuming and expensive and can be performed only by trained experts.^10,11,12^

A relatively new, emerging technique for analyzing VOCs in exhaled breath can be performed by electronic noses (e-noses). E-noses are portable, cheap, and easy-to-use diagnostic tests that are capable of producing rapid results. Detection of VOC patterns is possible because of binding of VOCs to sensors within e-noses. The binding of VOCs to these sensors generates an electrical response. This electrical response can then be measured. Different types of sensors are used in e-noses, with each sensor type having its own distinct advantages and disadvantages. Unlike GC-MS, e-noses are incapable of identifying individual VOCs. Furthermore, the accuracy of e-noses is affected by endogenous and exogenous factors, such as comorbidities, smoking, diet, body mass index, and ambient air.^13^ These factors may alter detected VOC patterns in exhaled breath.^14^ E-nose technologies analyzing exhaled breath have been extensively studied for oncologic indications and have been demonstrated to have promising results with high diagnostic accuracies.^15,16,17,18^

Several reviews^13,15,19^ have investigated the diagnostic accuracy of available e-noses for different indications. Reviewers have uniformly concluded that e-noses have the potential to become promising diagnostic tools in everyday clinical practice. Although these results are encouraging, at the time of this writing, no e-nose is being used in clinical practice to detect malignant tumors.

No systematic review is currently available that provides a pooled analysis of the diagnostic performance of e-noses for the detection of cancer. This systematic review with meta-analysis provides an overview of the diagnostic performance of all e-nose technologies currently used for cancer diagnosis in exhaled breath. Furthermore, this review identifies current obstacles and limitations found in this field of research.

## Methods

### Search Strategy

On January 7, 2021, an electronic search was performed in the PubMed and Embase databases. Keywords such as *lung diseases*, *gastrointestinal diseases*, *neoplasms*, *e-nose*, *electronic nose*, *volatile organic compounds*, *diagnosis*, *sensitivity and specificity*, and *ROC curve* were used as search terms, combined using AND/OR operators. The full search strategy can be found in eTable 1 in the Supplement. This study followed the Preferred Reporting Items for Systematic Reviews and Meta-analyses (PRISMA) reporting guideline.

**Figure 1.**
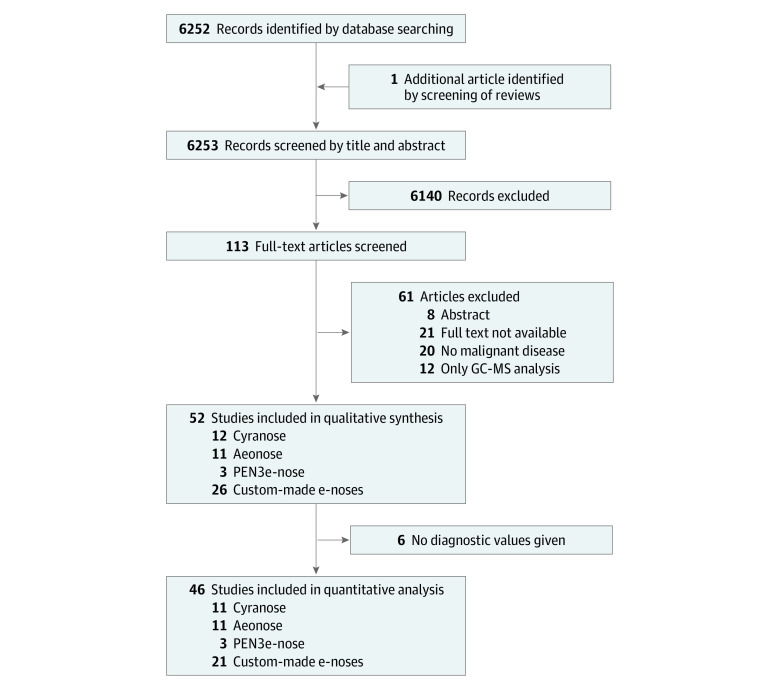
Identification, Screening, Eligibility, and Selection Process e-Nose indicates electronic nose; GC-MS, gas chromatography–mass spectrometry.

A total of 6253 articles were found. All articles were screened for eligibility by reading the title and abstract. A total of 113 articles were screened for eligibility by 2 independent reviewers (M.H.M.C.S. and Z.A.-D.) who read the full text. Diagnostic studies that met the following inclusion criteria were included: (1) use of e-nose technology, (2) detection of cancer, and (3) analysis of exhaled breath. The exclusion criteria were (1) studies published before 2000, (2) studies not performed in humans, (3) studies not performed in adults, (4) studies that only analyzed biofluids (including urine, blood, and/or feces), and (5) studies that exclusively used GC-MS to analyze breath samples. A total of 52 articles were deemed eligible for inclusion. Figure 1 shows the flow diagram of the identification, screening, eligibility, and selection process using the PRISMA guidelines. The following information was gathered independently by 2 reviewers (M.H.M.C.S. and Z.A.-D.) and tabulated from the articles: author, type of cancer, cancer stage, country of publication, year of publication, sample size, type of e-nose, reference test, method of data analysis, control groups, sensitivity, specificity, accuracy, area under the curve, true-positive results, false-positive results, true-negative results, and false-negative results.

### Quality Assessment

The methodologic quality of the selected studies was assessed by a modified version of the Quality Assessment of Diagnostic Studies 2 tool (QUADAS-2).^20^ This modified version was previously constructed by Hanna et al^1^ to improve the quality assessment of feasibility studies. Specific changes to the QUADAS-2 tool were focused on the importance given to the inclusion of benign conditions and healthy controls, internal and/or external validation of results, assessment before therapeutic intervention, and reproducibility of the chosen index test. Quality assessment was performed independently by 2 reviewers (M.H.M.C.S. and Z.A.-D.). Any discrepancies were resolved in a consensus meeting between these 2 reviewers. The full version of the modified QUADAS-2 can be found in eTable 2 in the Supplement.

The quality of the evidence was assessed by the Rational Clinical Examination Levels of Evidence scale.^21^ Quality of evidence is scored from 1/A (high quality) to 5/C (low quality). The full rating scale with a definition of the level of evidence scores is provided in eTable 3 in the Supplement.

### Statistical Analysis

A meta-analysis of all e-noses reporting diagnostic performance values was conducted on 46 studies, which included Aeonose (n = 11),^18,22,23,24,25,26,27,28,29,30,31^ Cyranose 320 (n = 11),^16,17,32,33,34,35,36,37,38,39,40^ PEN3 (n = 3),^41,42,43^ and custom-made e-noses (n = 21).^6,8,9,44,45,46,47,48,49,50,51,52,53,54,55,56,57,58,59,60,61^ Six studies^62,63,64,65,66,67^ did not report diagnostic performance values and, therefore, could not be included in the meta-analysis.

Pooled estimates of diagnostic accuracy were calculated using bivariate generalized mixed-effects regression models to account for the negative correlation between sensitivity and specificity.^68^ Sensitivity and specificity are estimated with their respective CIs and prediction in the receiver operating characteristic curve. A scatterplot using standardized predicted random effects (standardized level 2 residuals) was used to check for outliers. A spike plot was used for investigating particularly influential studies using Cook distance. Outliers and influential studies were excluded, and a meta-analysis of all available e-noses was performed again.

The Deeks funnel plot asymmetry test was performed to test for publication bias. A 2-sided *P* < .10 was assumed to be statistically significant.^69^ To quantify statistical heterogeneity among pooled studies, the *I*^2^ index was used. Stata software, version 17 (StataCorp LLC) was used to pool data, and the statistical package MIDAS was used for bivariate meta-analysis.^70^

Because of heterogeneity in used sensors, analytical methods, and sampling techniques, separate meta-analyses were performed for the Aeonose and Cyranose 320 to determine the individual diagnostic accuracy of these 2 e-noses. Furthermore, separate pooled analyses were performed for lung cancer (LC), colorectal cancer (CRC), and head and neck cancer (HNC) and for advanced and early tumor stages to determine the diagnostic accuracy of e-noses in detecting different cancer types and stages. No separate analysis was performed for other cancer types and the PEN3 e-nose because of an insufficient number of studies (n < 4) to perform bivariate meta-analysis.

Additional pooled analysis of studies with a low or unclear risk of bias in the patient selection domain of the QUADAS-2 tool was performed to assess the influence of studies with a high risk of bias in patient selection on the diagnostic accuracy. Similar pooled analyses were performed for 3 other QUADAS-2 domains: index test, reference test, and flow and timing.

## Results

### Study Characteristics

The literature search identified 52 publications^6,16,17,18,22,67^ that met the inclusion criteria with a total of 3677 patients with cancer across all studies. All studies were feasibility studies. The most commonly used e-noses were the Cyranose 320 (n = 12),^16,17,32,33,34,35,36,37,38,39,40,66^ Aeonose (n = 11),^18,22,23,24,25,26,27,28,29,30,31^ and PEN3 (n = 3).^41,42,43^ Furthermore, a large heterogeneous group of custom-made e-noses were used (n = 26).^6,8,9,44,45,46,47,48,49,50,51,52,53,54,55,56,57,58,59,60,61,62,63,64,65,67^ The most commonly used sensors were metal oxide sensors. Aeonose, PEN3, and several e-nose prototypes use metal oxide sensors to detect VOCs. Other sensor types reported were quartz microbalance sensors, conducting polymers, and a variety of nanomaterial-based sensors. Characteristics of the studies, including used sensor types, are summarized in the Table.

**Table.** Characteristics and Outcomes of All Studies Included in the Qualitative Analysis

Source	Cancer type/stage	Patients with cancer, No.	Controls	Model	Sensitivity, %	Specificity, %	AUC, %	Accuracy, %	eNose	Statistical method	Quality of evidence^a^
Altomare et al,^41^ 2016	CRC/primarily advanced stage	15	HCs (n = 15), benign (n = 15)	CRC (n = 15) vs HCs (n = 10)	93	10	NR	38	PEN3: 10 MOS	PNN	4/C
Amal et al,^6^ 2014	OC/mixed	48	Benign (n = 86), HCs (n = 48)	OC (n = 48) vs HC and benign (n = 134)	71	71	NR	71	Prototype: 9 nanomaterial sensors (GNP and SWCNTs)	DFA	4/C
Amal et al,^8^ 2015	CRC/primarily early stage	65	Benign (n = 22), HCs (n = 122)	CRC (n = 16) vs benign (n = 16)	94	88	NR	91	Prototype: 6 nanomaterial sensors (GNP and SWCNTs)	DFA	4/C
Amal et al,^44^ 2016	GC/primarily advanced stage	99	Benign (n = 385)	GC (n = 30) vs OLGIM 0-IV (n = 95)	73	98	NR	92	Prototype: 8 nanomaterial sensors (GNP and SWCNTs)	DFA	4/C
Barash et al,^62^ 2015	BC/NR	169	Benign (n = 52), HCs (n = 30)	BC vs benign and HCs (n = 140)	84	80	90	83	Prototype: 40 nanomaterial sensors (GNP and SWCNTs)	DFA	4/C
Broza et al,^45^ 2012	LC/early stage	12	Benign (n = 5)	LC (n = 12) vs benign (n = 5)	100	80	NR	94	Prototype: 25 nanomaterial sensors (GNP and PtNPs)	DFA	4/C
Capuano et al,^63^ 2015	LC/NR	20	Benign (n = 10)	LC (n = 20) vs benign (n = 10)	NR	NR	NR	93	LibraNose: 8 QMB sensors	PLS-DA	4/C
Chapman et al,^32^ 2012	MPM/early stage	20	HCs (n = 42), benign (n = 18)	MPM (n = 10) vs HCs (n = 32)	90	91	NR	95	Cyranose 320: 32 conducting polymer sensors	PCA	4/C
Chen et al,^46^ 2021	LC/primarily advanced stage	101	HCs (n = 134)	LC (n = 101) vs HCs (n = 134)	95.6	91.1	NR	93.6	Prototype: 11 sensors: MOS, EGS, HWGS CCGS	KPCA-XGBoost	4/C
Chen Q et al,^47^ 2020	LC/NR	48	HC (n = 48)	LC (n = 24) vs HCs (n = 25)	96	96	100	NR	Prototype: GO sensor	LDA	4/C
de Kort et al,^23^ 2018	LC/primarily advanced stage	144	HCs and suspected (n = 146)	NSCLC (n = 144) vs HCs and suspected (n = 146)	94.4	32.9	76	NR	Aeonose: 3 MOS	ANN–cross-validation (Aethena)	4/C
de Kort et al,^22^ 2020	LC/primarily advanced stage	138	HCs (n = 84), suspected (n = 59)	NSCLC (n = 138) vs non-LC (n = 143)	94.2	44.1	75	NR	Aeonose: 3 MOS	ANN (Aethena)	4/C
de Vries et al,^64^ 2015	LC/primarily advanced stage	31	Benign (n = 68), HCs (n = 45)	LC (n = 31) vs benign (n = 31)	NR	NR	95	88	SpiroNose: 4 MOS sensors	PCA	4/C
Di Natale et al,^65^ 2003	LC/mixed	35	HCs (n = 18)	LC (n = 35) and HCs (n = 18)	NR	NR	NR	94	Prototype: 8 QMB sensors	PLS-DA	4/C
Diaz de Leon-Martinez et al,^33^ 2020	BC/mixed	262	HCs (n = 181)	BC (n = 262) vs HCs (n = 181)	100	100	NR	98.7	Cyranose 320: 32 conducting polymer sensors	CDA	4/C
Dragonieri et al,^66^ 2009	LC/mixed	10	Benign (n = 10), HCs (n = 10)	LC (n = 10) vs benign (n = 10)	NR	NR	NR	85	Cyranose 320: 32 conducting polymer sensors	PCA	4/C
Dragonieri et al,^16^ 2012	MPM/primarily early stage	13	HCs (n = 13), AEx (n = 13)	MPM (n = 13) vs AEx (n = 13)	92	86	92	81	Cyranose 320: 32 conducting polymer sensors	CVA	4/C
Gasparri et al,^48^ 2016	LC/primarily early stage	70	HCs (n = 76)	LC (n = 21) and HCs (n = 20)	81	100	87	NR	Prototype: 8 QMB sensors	PLS-DA	4/C
Gruber et al,^9^ 2014	HNSCC/mixed	22	Benign (n = 21), HCs (n = 19)	HNSCC (n = 22) and HCs (n = 19)	77	90	NR	83	Prototype:6 nanomaterial sensors (GNP and SWCNTs)	DFA	4/C
Hakim et al,^49^ 2011	HNSCC/primarily advanced stage	22	HCs (n = 40)	HNSCC (n = 16) and HCs (n = 24)	92	100	NR	95	NA-NOSE: 5 GNP sensors	PCA	4/C
Herman-Saffar et al,^34^ 2018	BC/early stage	48	HCs (n = 45)	BC (n = 33) vs HCs (n = 32)	48	62	NR	55	Cyranose 320: 32 conducting polymer sensors	FE-ANN (1000-fold)	4/C
Huang et al,^35^ 2018	LC/primarily early stage	56	Nontumor controls (n = 188)	LC (n = 12) vs non-LC (n = 29)	83	86	NR	85	Cyranose 320: 32 conducting polymer sensors	SVM (external validation)	4/C
Hubers et al,^36^ 2014	LC/primarily advanced stage	20	Benign (n = 31)	LC (n = 18) vs benign (n = 8)	94	13	66	NR	Cyranose 320: 32 conducting polymer sensors	PCA	4/C
Kononov et al,^50^ 2019	LC/mixed	65	HCs (n = 53)	LC (n = 65) vs HCs (n = 53)	95	100	95.6	97.2	Prototype: 6 MOS	LRA without PCA decomposition	4/C
Krauss et al,^24^ 2020	LC/primarily advanced stage	120	Benign (n = 197), HCs (n = 33)	LC (n = 91) vs HCs (n = 33)	84	97	92	73	Aeonose: 3 MOS	ANN (Aethena)	4/C
Lamote et al,^37^ 2017	MPM/NR	14	HCs (n = 16), AEx (n = 19), benign ARD (n = 15)	MPM (n = 11) vs benign (n = 27)	82	55	75	74	Cyranose 320: 32 conducting polymer sensors	PCA	4/C
Leja et al,^51^ 2021	GC/primarily advanced stage	94	HCs (n = 180)	GC (n = 31) vs HCs (n = 65)	87	85	92	85	SniffPhone (SGNPs)	LDA	4/C
Leunis et al,^52^ 2014	HNSCC/primarily advanced stage	36	Benign (n = 23)	HNSCC (n = 36) vs benign (n = 23)	90	80	89	85	Prototype:12 MOS	LRA	4/C
Li et al,^53^ 2017	LC/NR	24	HCs (n = 23), benign (n = 5)	LC (n = 24) vs non-LC (n = 28)	92	92	NR	92	Prototype: 14 sensors, MOS HWG, CCGS, EGS	LDA (fuzzy 5-NN)	4/C
Li et al,^67^ 2020	LC/primarily advanced stage	115	HCs (n = 153)	LC (n = 115) vs HCs (n = 153)	NR	NR	87	NR	Prototype: 10 sensors, MOS HWG, CCGS, EGS	LDA combined with PCA, Fast ICA, NMF, and Kbest	4/C
Liu et al,^54^ 2021	LC/advanced stage	98	HCs (n = 116)	LC (n = 98) vs HCs (n = 116)	95.3	97.2	NR	96.1	Prototype: 11 sensors, MOS HWG, CCGS, EGS	PCA-SVE	4/C
Machado et al,^17^ 2005	LC/primarily advanced stage	14	Benign (n = 62)	LC (n = 14) vs non-LC (n = 62)	71	92	NR	85	Cyranose 320: 32 conducting polymer sensors	SVM	4/C
Marzorati et al,^55^ 2019	LC/early stage	6	HCs (n = 10)	LC (n = 6) vs HCs (n = 10)	86	100	NR	94	Prototype: 4 MOS	ANN (LOOCV)	4/C
Mohamed et al,^25^ 2021	OSCC/primarily advanced stage	49	HCs (n = 35)	OSCC (n = 49) vs HCs (n = 35)	88	71	86	81	Aeonose: 3 MOS	ANN (Aethena)	4/C
Mohamed et al,^42^ 2019	LC/primarily advanced stage	50	Benign (n = 50)	LC (n = 28) vs benign (n = 20)	93	90	NR	92	PEN3: 10 MOS	PCA and ANN	4/C
Peled et al,^56^ 2012	LC/mixed	53	Benign (n = 19)	LC (n = 50) vs benign (n = 19)	86	96	99	88	Prototype: 18 nanomaterial sensors (GNPs and SWCNTs)	DFA	4/C
Raspagliesi et al,^43^ 2020	OC/primarily advanced stage	86	HCs (n = 114), benign (n = 51)	OC (n = 28) vs HCs and benign (n = 55)	82	93	NR	87	PEN3: 10 MOS	KNN (strict prediction)	4/C
Rocco et al,^57^ 2016	LC/advanced stage	23	HCs (n = 77)	LC (n = 23) vs HCs (n = 77)	86	95	87	NR	BIONOTE: 7 Acoustic-mass sensors	PLS-DA	4/C
Schuermans et al,^26^ 2018	GC/NR	16	HCs (n = 28)	GC (n = 16) vs benign (n = 28)	81	71	NR	75	Aeonose: 3 MOS	ANN (Aethena)	4/C
Shehada et al,^58^ 2016	LC/primarily advanced stage	149	Benign (n = 56), HCs (n = 129)	LC n = 149 vs non-LC n = 56	89	75	NR	86	Prototype: SNS	DFA	4/C
Shlomi et al,^59^ 2017	LC/primarily advanced stage	89	Benign (n = 30)	LC (n = 16) vs benign (n = 30)	75	93	NR	87	Prototype: 40 nanomaterial sensors	DFA	4/C
Steenhuis et al,^27^ 2020	Recurrent CRC/primarily advanced stage	26	No recurrence (n = 36)	CRC positive (n = 26) vs CRC negative (n = 36)	88	75	86	81	Aeonose: 3 MOS	ANN (Aethena)	4/C
Tan et al,^60^ 2016	LC/advanced stage	12	Benign (n = 12), HCs (n = 13)	LC (n = 12) vs non-LC (n = 25)	83	88	86	NR	Prototype: chemiresistor-based alkane sensor	MANOVA	4/C
Tirzite et al,^38^ 2017	LC/NR	165	HCs (n = 79), benign (n = 91)	LC (n = 45) vs HCs (n = 16)	98	69	NR	90	Cyranose 320: 32 conducting polymer sensors	SVM	4/C
Tirzite et al,^39^ 2019	LC/advanced stage	252	Benign and HCs (n = 223)	LC (n = 119) vs non-LC (n = 91)	95.8	92.3	NR	NR	Cyranose 320: 32 conducting polymer sensors	LRA	4/C
van de Goor et al,^28^ 2018	LC/primarily advanced stage	52	HCs (n = 93)	LC (n = 8) vs HCs (n = 14)	88	86	NR	86	Aeonose: 3 MOS	ANN (Aethena)	4/C
van de Goor et al,^18^ 2019	Recurrent HNSCC/mixed	20	No recurrence (n = 20)	HNSCC recurrence positive (n = 20) vs HNSCC recurrence negative (n = 20)	85	80	85	83	Aeonose: 3 MOS	ANN (Aethena)	4/C
van de Goor et al,^29^ 2020	HNSCC/mixed	91	HCs (n = 72)	HNSCC (n = 91) vs HCs (n = 72)	79	63	75	72	Aeonose: 3 MOS	ANN (Aethena)	4/C
van Keulen et al,^30^ 2020	CRCr/mixed	70	Benign (n = 234), HCs (n = 128)	CRC (n = 62) vs HCs (n = 104)	95	64	84	NR	Aeonose: 3 MOS	ANN (Aethena)	4/C
Waltman,^31^ 2020	PC/primarily early stage	32	Benign (n = 53)	PC (n = 32) and benign (n = 53)	84	70	79	77	Aeonose: 3 MOS	ANN (Aethena)	4/C
Xu et al,^61^ 2013	GC/mixed	37	Benign (n = 93)	GC (n = 37) vs benign (n = 93)	89	90	NR	90	Prototype:14 nanomaterial sensors (GNP and SWCNTs);	DFA	4/C
Yang et al,^40^ 2021	BC/NR	351	HCs (n = 88)	BC (n = 351) vs HCs (n = 88)	86	97	99	91	Cyranose 320: 32 conducting polymer sensors	Random forest	4/C

Abbreviations: AEx, asymptomatic former asbestos exposure; ANN, artificial neural network; ARD, asbestos-related disease; BC, breast cancer; CCGS, catalytic combustion gas sensor; CDA, canonical discriminant analysis; CRC, colorectal cancer; DFA, discriminate function analysis; EGS, electrochemical gas sensor; FE, feature extraction; GC, gastric cancer; GNP, gold nanoparticles; GO, graphene oxide; HCs, healthy controls; HNSCC, head and neck squamous cell carcinoma; HWGS, hot wire gas sensor; ICA, independent component analysis; KNN, K-nearest neighbors; KPCA, kernel principal component analysis; LC, lung cancer; LDA, linear discriminant analysis; LOOCV, leave-one-out cross-validation; LRA, logistic regression analysis; MANOVA, multivariate analysis of variance; MOS, metal oxide sensor; MPM, malignant pleural mesothelioma; NMF, nonnegative matrix factorization; NN, neural network; NR, not reported; NSCLC, non–small cell lung carcinoma; OC, ovarian cancer; OLGIM, operative link on gastric intestinal metaplasia; OSCC, oral squamous cell carcinoma; PCA, principal component analysis; PLS-DA, partial least-squares discriminant analysis; PC, prostate cancer; PNN, probabilistic neural network; PtNP, platinum nanoparticles; QMB, quartz microbalance; SNS, silicon nanowire sensor; SVE, statistical volume element; SVM, support vector machines; SWCNT, single-walled carbon nanotubes; XGBoost, eXtreme Gradient Boosting.

^a^ Quality of evidence rating conducted by the Rational Clinical Examination Levels of Evidence scale (for full details of the scale, see eTable 3 in the Supplement).

Fourteen studies^18,22,23,24,25,26,27,28,29,30,31,51,57,64^ used an e-nose that enabled direct breath sampling. Eleven of these studies^18,22,23,24,25,26,27,28,29,30,31^ used the Aeonose, whereas other studies used Spironose (Breathomix),^64^ Bionote (Campus Bio-Medico University),^57^ and SniffPhone (Technion Institute of Technology).^51^

Thirty-four studies^6,8,9,16,18,32,35,36,37,38,39,40,41,42,43,44,45,46,48,49,52,53,54,55,56,58,59,61,62,63,64,65,66,67^ used sampling bags to collect exhaled breath. Of these, 19 studies^6,16,35,36,37,40,41,46,48,52,53,54,55,58,61,62,63,66,67^ used Tedlar bags, 6 studies^9,17,45,49,56,58^ used Mylar bags, 3 studies^8,44,59^ used GasAmpler bags, and 6 studies^32,38,39,42,43,65^ used other or unspecified bags.

A variety of statistical methods were used to analyze VOC patterns in exhaled breath. Artificial neural networks were the most frequently reported analytical method. Other examples of reported analytical methods were discriminate function analysis, principal component analysis, partial least squares discriminant analysis, and canonical discriminant analysis. The Table provides an overview of the analytical methods reported.

The number of included patients with cancer ranged from 10 to 351. Types of cancer studied were lung (n = 28), head and neck (n = 5), gastric (n = 4), breast (n = 4), colorectal (n = 4), mesothelioma (n = 3), oral cavity (n = 2), ovarian (n = 1), and prostate (n = 1) (Table). Most studies compared patients with cancer with a control group of healthy volunteers and/or patients with benign disease. Most studies did not perform any diagnostic tests to exclude malignant tumors in healthy volunteers. Histopathological analysis was used for diagnostic confirmation of malignant tumors in 50 studies; 2 studies^47,65^ did not report the method used for diagnostic confirmation of malignant tumors. Studies included patients with early and advanced tumor stages, often with various histological types. Tumor stage was not reported in 8 studies.^26,37,38,40,45,47,53,63^

Consideration and reporting of exogenous and endogenous factors during exhaled breath collection differed largely among studies. A variety of measures were implemented to reduce possible effects of confounding factors on exhaled VOCs. Fasting before breath sampling was performed in 30 studies (58%).^6,8,9,16,32,33,35,36,41,43,44,45,46,47,48,50,51,52,53,54,55,57,58,59,60,61,62,65,66,67^ Duration of fasting differed largely among these studies. Cessation of smoking was applied in 24 studies (46%).^8,9,24,33,35,36,40,43,44,45,47,48,50,51,53,54,55,57,58,59,60,61,66,67^ Minimum duration of cessation also differed among studies. In 5 studies (10%),^32,51,53,61,67^ patients were asked to rest for a certain amount of time before breath sampling. Other measures to reduce potential exogenous confounding factors included rinsing of the mouth, cessation of perfume, cessation of pungent food, and use of inspiratory VOC filters. Furthermore, a variety of comorbidities were considered as having a potential confounding effect on VOCs in exhaled breath. In most studies, patients with certain comorbidities were excluded from participation. Examples of several endogenous and exogenous factors and measures to reduce their influence are given in eTable 4 in the Supplement.

### Diagnostic Accuracy

**Figure 2.**
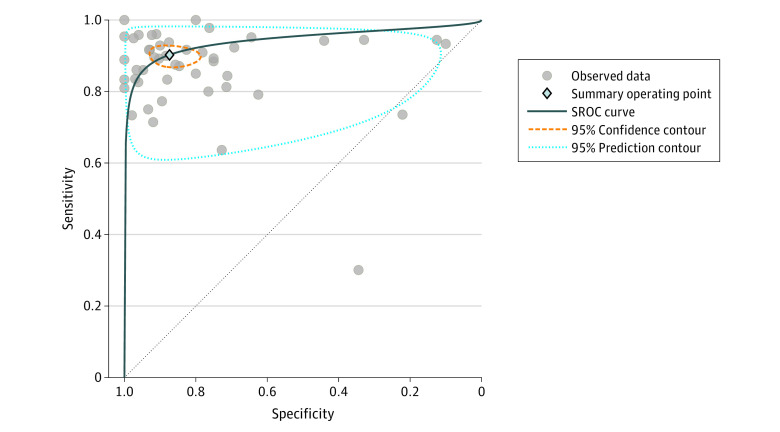
Summary Receiver Operating Characteristic (SROC) Curve Analysis of All Electronic Noses For the summary operating point, sensitivity was 0.90 (95% CI, 0.88-0.92) and specificity was 0.87 (95% CI, 0.81-0.92). For the SROC curve, the area under the curve was 0.94 (95% CI, 0.92-0.95).

**Figure 3.**
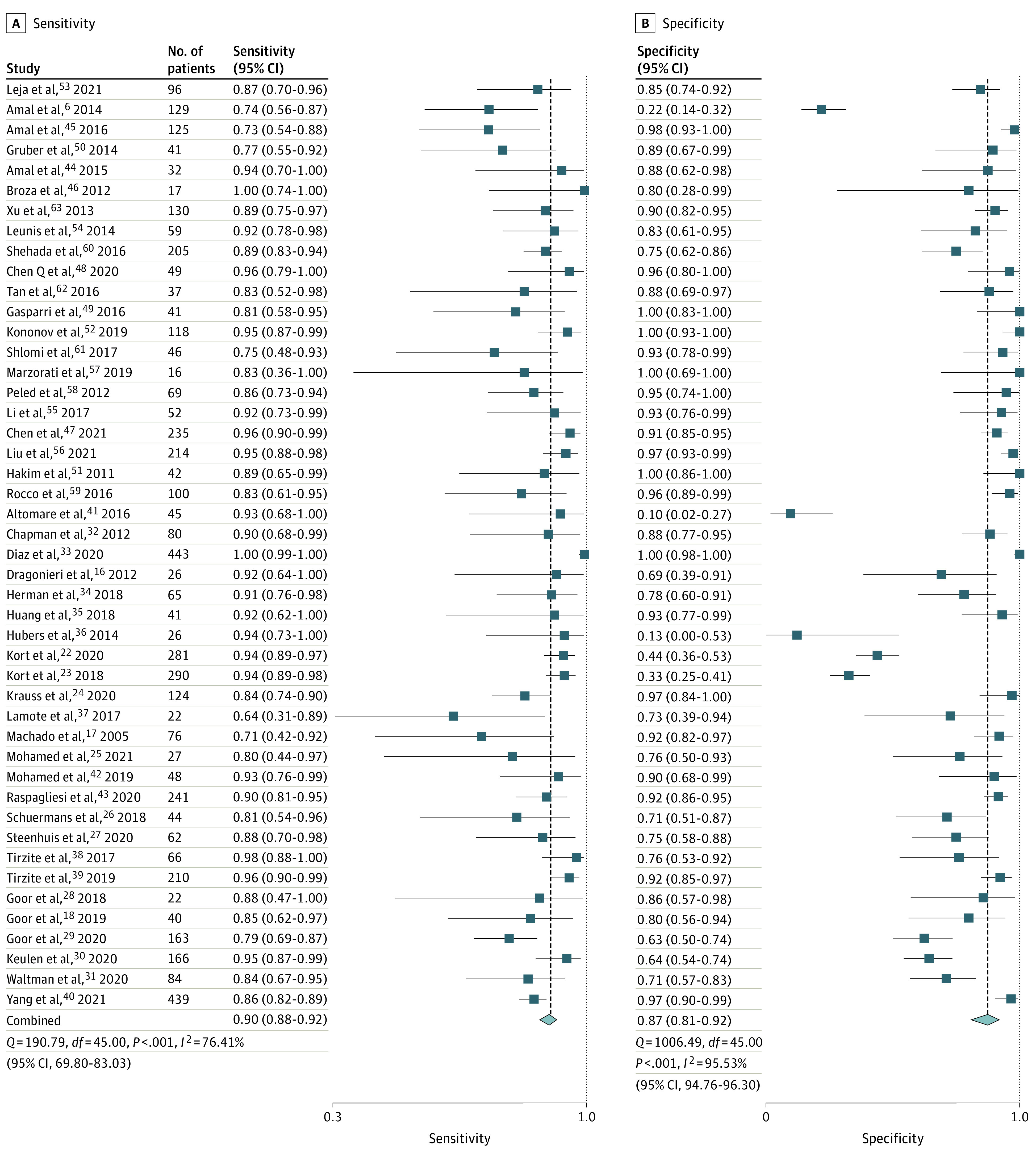
Pooled Sensitivity and Specificity Analyses of All Electronic Noses

Sensitivity of all e-noses ranged from 48.3% to 95.8% for the detection of cancer. Specificity ranged from 10.0% to 100.0%. Pooled receiver operating characteristic analysis of all e-noses resulted in a pooled area under the curve of 94% (95% CI, 92%-96%), sensitivity of 90% (95% CI, 88%-92%), and specificity of 87% (95% CI, 81%-92%) (Figure 2 and Figure 3). Pooled studies had an *I*^2^ index of 76.41% for sensitivity and 95.53% for specificity, which corresponds to a high statistical heterogeneity. Additional meta-analysis after exclusion of outliers and influential studies (eFigure 1 in the Supplement) showed similar results, with a pooled sensitivity of 90% (95% CI, 87%-91%) and a pooled specificity of 89% (95% CI, 84%-92%), indicating limited influence of outliers and influential studies on the results (eFigure 2 in the Supplement). The Deeks funnel plot asymmetry test showed a significant funnel plot asymmetry (intercept, 7.01%; 95% CI, 5.01%- 9.01%; *P* = .02) (eFigure 3 in the Supplement).

A separate pooled analysis for the Cyranose 320 demonstrated its ability to detect cancer with a sensitivity of 93% (95% CI, 85%-97%; *I*^2^ = 83.28%) and a specificity of 89% (95% CI, 72%-96%; *I*^2^ = 89.80%) (eFigure 4 in the Supplement). Separate pooled analysis for the Aeonose showed a sensitivity of 88% (95% CI, 83%-92%; *I*^2^ = 71.92%) and a specificity of 70% (95% CI, 57%-80%; *I*^2^ = 90.25%) (eFigure 5 in the Supplement).

Separate pooled analyses of LC, HNC, and CRC resulted in a sensitivity of 92% (95% CI, 89%-94%; *I*^2^ = 80.87%) and a specificity of 91% (95% CI, 83%-95%; *I*^2^ = 96.07%) for LC, a sensitivity of 85% (95% CI, 77%-90%; *I*^2^ = 50.49%) and a specificity of 85% (95% CI, 68%-94%; *I*^2^ = 82.86%) for HNC, and a sensitivity of 93% (95% CI, 87%-97%; *I*^2^ = 0.00%) and a specificity of 59% (95% CI, 24%-87%; *I*^2^ = 92.56%) for CRC (eFigures 6, 7, and 8 in the Supplement, respectively). Additional sensitivity analysis for advanced and early tumor stage resulted in similar accuracies, with a sensitivity of 90% (95% CI, 87%-93%; *I*^2^ = 75.11%) and a specificity of 86% (95% CI; 75%-93%; *I*^2^ = 96.20%) for advanced stage tumors and a sensitivity of 89% (95% CI, 83%-93%; *I*^2^ = 0.00%) and a specificity of 86% (95% CI, 77%-92%; *I*^2^ = 57.94%) for early stage tumors (eFigures 9 and 10 in the Supplement).

Pooled analysis of studies with a low or unclear risk of bias in the patient selection domain of the QUADAS-2 tool resulted in a sensitivity of 91% (95% CI, 0.87%-0.94%; *I*^2^ = 83.56%) and a specificity of 87% (95% CI, 0.77%-0.93%; *I*^2^ = 96.69%) (eFigure 11 in the Supplement). Separate analyses of the other QUADAS-2 risk of bias domains resulted in similar diagnostic accuracies (eFigures 12-14 in the Supplement).

### Quality Assessment

The results of the quality assessment using QUADAS-2 are shown in eTables 5 and 6 in the Supplement. Overall, a high risk of bias was found in most studies. Regarding the use of the e-nose (index test), a total of 43 studies^6,16,17,18,22,23,24,25,26,27,28,29,30,31,32,33,34,35,36,37,38,39,40,41,42,43,45,47,48,49,50,52,54,55,56,57,58,59,60,63,64,65,66^ (83%) had a high risk of bias, mainly because of a lack of standardization and reproducibility of methods. Regarding flow and timing, a high risk of bias was found in 28 studies^16,17,22,23,24,25,28,29,31,32,34,36,40,45,46,48,49,52,53,54,55,56,58,60,63,64,65,67^ (54%). The main cause of the high risk of bias for flow and timing was insufficient consideration of endogenous and exogenous factors. Regarding patient selection, a high risk of bias was found in 21 studies^6,16,28,29,31,34,35,40,42,46,48,52,53,55,58,61,63,64,65,66,67^ (40%). The main cause of the high risk of bias for patient selection was the lack of matching patient groups. The risk of bias was lowest for the reference standard criterion, with only 2 studies^40,46^ (4%) showing high risk of bias. Other causes of bias included the absence of a validation set and the failure to report a time interval between the index test and the reference test. Furthermore, a significant concern was raised regarding the applicability of the study design to the study question as a result of inadequate patient selection criteria. This concern resulted in a high-applicability concern for patient selection criteria in 22 studies^9,16,17,22,23,24,28,29,35,38,39,43,46,48,50,51,58,63,64,65,66,67^ (42%). No significant concerns were found regarding the applicability of the index test and reference test to the study question. All studies had a Rational Clinical Examination Level of Evidence rating of 4/C (Table; eTable 3 in the Supplement).

## Discussion

We conducted a systematic review and meta-analysis of studies that investigated the diagnostic accuracy of e-noses in detecting cancer from exhaled breath samples. Pooled analysis of all e-noses demonstrated a high diagnostic accuracy of e-noses for the detection of cancer, with a pooled sensitivity of 90% and specific of 87%. The Aeonose and Cyranose 320 demonstrated similarly high diagnostic accuracies in detecting cancer. Furthermore, e-noses showed a high diagnostic accuracy in detecting LC, HNC, and CRC, although e-noses had a relatively low specificity in detecting CRC compared with LC and HNC. The high diagnostic accuracy of e-noses found in this study is in line with results from previous reviews^2,19^ that demonstrated similar diagnostic performance in detecting cancer in exhaled breath. Although these results are promising, they should be interpreted with caution because of high heterogeneity among studies, a high risk of bias found in most studies, and the potential presence of publication bias. Before e-noses can be implemented in daily clinical practice, several important issues must be addressed.

Various analytical methods, such as machine learning, were used to analyze VOCs in exhaled breath. Most studies here did not adequately explain how these analytical techniques were used, which means these e-nose studies have limited reproducibility. Because of the complex nature of machine learning, in their review, Sar et al^13^ recommended that an open-source database be constructed wherein data and know-how about machine learning could be freely and fairly exchanged. Such a development would improve the reproducibility and standardization of the analytical methods used in e-nose research.

One of the main technical disadvantages inherent to e-noses is sensor drift, which is defined as a gradual change in sensor output, independent of changes in measured sample or input. This change may lead to a gradual decrease in instrument sensitivity, which could lead to a false diagnosis.^19,71^ Sensor drift is caused by a variety of factors. Although several techniques have been proposed and developed to compensate for sensor drift,^71,72^ few studies in this review mentioned implementing these techniques. Furthermore, it is important to note that e-nose sensors have a limited sensor life, after which their sensitivity decreases.^73^ A potential solution to limited sensor life and sensor drift could be the use of disposable sensors.^19^

In concordance with several other reviews,^13,15,19^ a general lack of standardization was observed in study design and reporting of methods and results. Studies differed substantially with regard to patient selection criteria, consideration of endogenous and exogenous factors, exhaled breath collection, and analysis. Differences in study design might be the cause of the high statistical heterogeneity found in the pooled analysis. Separate analysis of the Cyranose 320 and Aeonose did not result in a significant reduction of statistical heterogeneity. This finding suggests that technological differences among e-noses are not the main cause of the statistical heterogeneity found. A lack of standardization in study design and reporting resulted in an overall low methodologic quality, with most studies having a risk of bias. Recently, Hanna et al^1^ constructed a comprehensive framework for the standardization of VOC-based studies. Conducting e-nose studies in a standardized manner using such a framework would improve the overall quality of e-nose research.

Many of the studies did not report on factors that could influence VOC analysis. Several endogenous and exogenous factors may affect breath profile, such as smoking, comorbidities, diet, age, sex, body mass index, and medication.^74^ However, the influence of such factors on breath prints needs to be investigated further. The reported measures to minimize the effects of these factors varied among studies. Insufficient consideration of these factors limits the validity and generalizability of results. Future research should thus focus on conducting more preclinical studies to investigate the effects of potential confounders on patients’ breath prints.

Most studies did not perform any diagnostic tests to exclude malignant tumors in healthy volunteers, so it is possible that healthy controls had an underlying malignant tumor. Most studies were feasibility studies with relatively small sample sizes used for internal validation, and although calculating correct sample sizes for internal validation fell outside the scope of this review, an extensive guide for sample size calculations has recently been published.^75^ Adequately powered external validation studies to confirm the results of these feasibility studies are rarely conducted. Because e-noses are particularly sensitive to changes in endogenous and exogenous factors, the diagnostic accuracy may vary among different research settings and patient groups. Therefore, it is important to conduct large, multicenter external validation studies to investigate the generalizability and reproducibility of the results in different research settings and different patient groups.

Separate analyses of LC, HNC, and CRC studies demonstrated that e-noses have a slightly higher diagnostic accuracy in detecting LC, with a pooled sensitivity of 92% and a pooled specificity of 91%. However, the diagnostic accuracy of the other cancer types was similarly high, with the exception of CRC, which had a lower specificity compared with other cancer types. Future studies should further investigate which cancer type could benefit most from the use of e-noses using exhaled breath.

### Limitations

This study has limitations. Substantial heterogeneity was observed among studies, making the interpretation of the results of the meta-analysis difficult. Furthermore, significant funnel plot asymmetry was detected, which might indicate the presence of publication bias. Although funnel plots are widely used to investigate publication bias, funnel plot asymmetry might have other possible causes, such as poor methodologic design, heterogeneity, and chance.^76^ In addition, a large number of studies had a high risk of bias, although sensitivity analyses by excluding studies with a high risk of bias had a limited effect on the pooled diagnostic accuracy.

All studies included here were diagnostic cross-sectional studies with a case-control study design. This study design is not an accurate representation of daily practice in which consecutive patients would undergo a diagnostic test. Furthermore, a significant number of studies primarily included patients with advanced stages of cancer. The discriminatory ability of e-noses for patients with early and advanced stages of disease is still under investigation. This review did not include studies that used e-noses to analyze biofluids, such as urine and blood. There is a possibility that biofluids could potentially be more suitable than exhaled breath for VOC analysis for certain cancers.

## Conclusions

The results of this systematic review with meta-analysis indicate that e-noses have a relatively high diagnostic accuracy in the detection of cancer in exhaled breath. However, the existing e-nose studies generally consist of feasibility studies with small sample sizes, a lack of standardization, and a high risk of bias. Thus, there is a need for adequately powered, multicenter external validation studies to establish the potential of e-noses in the diagnostic workup of cancer. Before clinical implementation can be realized, the lack of standardization and reproducibility in the field of e-nose research must be addressed.

## Notes

Supplement. **eTable 1.** Full Electronic Search Strategy Performed in the PubMed and Embase Databases **eTable 2.** Modified QUADAS-2 Assessment Tool **eTable 3.** The Rational Clinical Examination Levels of Evidence Scale **eTable 4.** Examples of Reported Confounding Factors and Measures to Reduce Influences **eTable 5.** Quality Assessment Using QUADAS-2 Tool: Summary of Risk of Bias and Concerns Regarding Applicability for Included Studies **eTable 6.** Quality Assessment Using QUADAS-2 Tool: Outcomes of Risk of Bias and Applicability Concerns Assessment for Individual Studies **eFigure 1.** Outlier and Influence Analysis of All e-Nose Studies **eFigure 2.** Pooled Analysis of All e-Nose Studies After Exclusion of Outliers **eFigure 3.** Publication Bias Analysis for All e-Nose Studies **eFigure 4.** Pooled Analysis of all Cyranose 320 Studies **eFigure 5.** Pooled Analysis of All Aeonose Studies **eFigure 6.** Pooled Analysis of All Lung Cancer Studies **eFigure 7.** Pooled Analysis of All Head and Neck Cancer Studies **eFigure 8.** Pooled Analysis of All Colorectal Cancer Studies **eFigure 9.** Pooled Analysis of All Advanced Cancer Stage Studies **eFigure 10.** Pooled Analysis of All Early Cancer Stage Studies **eFigure 11.** Pooled Analysis of All Studies After Exclusion of Studies With a High Risk of Bias on the Patient Selection Domain of the QUADAS-2 Tool **eFigure 12.** Pooled Analysis of All Studies After Exclusion of Studies With a High Risk of Bias on the Index Test Domain of the QUADAS-2 Tool **eFigure 13.** Pooled Analysis of All Studies After Exclusion of Studies With a High Risk of Bias on the Reference Standard Domain of the QUADAS-2 Tool **eFigure 14.** Pooled Analysis of All Studies After Exclusion of Studies With a High Risk of Bias on the Flow and Timing Domain of the QUADAS-2 Tool
