# Supplementary material for: Diagnostic Performance of Electronic Noses in Cancer Diagnoses Using Exhaled Breath: A Systematic Review and Meta-analysis
Source: JAMA Netw Open. 2022 Jun 29;5(6):e2219372. doi: 10.1001/jamanetworkopen.2022.19372 (PMC9244610; doi:10.1001/jamanetworkopen.2022.19372)
Supplement: Supplement. — eTable 1. Full Electronic Search Strategy Performed in the PubMed and Embase Databases eTable 2. Modified QUADAS-2 Assessment Tool eTable 3. The Rational Clinical Examination Levels of Evidence Scale eTable 4. Examples of Reported Confounding Factors and Measures to Reduce Influences eTable 5. Quality Assessment Using QUADAS-2 Tool: Summary of Risk of Bias and Concerns Regarding Applicability for Included Studies eTable 6. Quality Assessment Using QUADAS-2 Tool: Outcomes of Risk of Bias and Applicability Concerns Assessment for Individual Studies eFigure 1. Outlier and Influence Analysis of All e-Nose Studies eFigure 2. Pooled Analysis of All e-Nose Studies After Exclusion of Outliers eFigure 3. Publication Bias Analysis for All e-Nose Studies eFigure 4. Pooled Analysis of all Cyranose 320 Studies eFigure 5. Pooled Analysis of All Aeonose Studies eFigure 6. Pooled Analysis of All Lung Cancer Studies eFigure 7. Pooled Analysis of All Head and Neck Cancer Studies eFigure 8. Pooled Analysis of All Colorectal Cancer Studies eFigure 9. Pooled Analysis of All Advanced Cancer Stage Studies eFigure 10. Pooled Analysis of All Early Cancer Stage Studies eFigure 11. Pooled Analysis of All Studies After Exclusion of Studies With a High Risk of Bias on the Patient Selection Domain of the QUADAS-2 Tool eFigure 12. Pooled Analysis of All Studies After Exclusion of Studies With a High Risk of Bias on the Index Test Domain of the QUADAS-2 Tool eFigure 13. Pooled Analysis of All Studies After Exclusion of Studies With a High Risk of Bias on the Reference Standard Domain of the QUADAS-2 Tool eFigure 14. Pooled Analysis of All Studies After Exclusion of Studies With a High Risk of Bias on the Flow and Timing Domain of the QUADAS-2 Tool [file jamanetwopen-e2219372-s001.pdf]

Supplementary Online Content

Scheepers MHMC, Al-Difaie Z, Brandts L, Peeters A, van Grinsven B, Bouvy ND. Diagnostic performance of electronic noses in cancer diagnoses using exhaled breath: a systematic review and meta-analysis. *JAMA Netw Open*. 2022;5(6):e2219372. doi:10.1001/jamanetworkopen.2022.19372

eTable 1. Full Electronic Search Strategy Performed in the PubMed and Embase Databases

eTable 2. Modified QUADAS-2 Assessment Tool

eTable 3. The Rational Clinical Examination Levels of Evidence Scale

eTable 4. Examples of Reported Confounding Factors and Measures to Reduce Influences

eTable 5. Quality Assessment Using QUADAS-2 Tool: Summary of Risk of Bias and Concerns Regarding Applicability for Included Studies

eTable 6. Quality Assessment Using QUADAS-2 Tool: Outcomes of Risk of Bias and Applicability Concerns Assessment for Individual Studies

eFigure 1. Outlier and Influence Analysis of All e-Nose Studies

eFigure 2. Pooled Analysis of All e-Nose Studies After Exclusion of Outliers

eFigure 3. Publication Bias Analysis for All e-Nose Studies

eFigure 4. Pooled Analysis of all Cyranose 320 Studies

eFigure 5. Pooled Analysis of All Aeonose Studies

eFigure 6. Pooled Analysis of All Lung Cancer Studies

eFigure 7. Pooled Analysis of All Head and Neck Cancer Studies

eFigure 8. Pooled Analysis of All Colorectal Cancer Studies

eFigure 9. Pooled Analysis of All Advanced Cancer Stage Studies

eFigure 10. Pooled Analysis of All Early Cancer Stage Studies

eFigure 11. Pooled Analysis of All Studies After Exclusion of Studies With a High Risk of Bias on the Patient Selection Domain of the QUADAS-2 Tool

eFigure 12. Pooled Analysis of All Studies After Exclusion of Studies With a High Risk of Bias on the Index Test Domain of the QUADAS-2 Tool

eFigure 13. Pooled Analysis of All Studies After Exclusion of Studies With a High Risk of Bias on the Reference Standard Domain of the QUADAS-2 Tool

eFigure 14. Pooled Analysis of All Studies After Exclusion of Studies With a High Risk of Bias on the Flow and Timing Domain of the QUADAS-2 Tool

This supplementary material has been provided by the authors to give readers additional information about their work.

© 2022 Scheepers MHMC et al. *JAMA Network Open.*

eTable 1: Full electronic search strategy performed in the PubMed-, and Embase- databases.

#	Search
1.	Lung Diseases/
2.	Gastrointestinal Diseases/
3.	Neoplasms/
4.	lung disease$.ti,ab,kw.
5.	gastrointestinal disease$.ti,ab,kw.
6.	carcinom$.ti,ab,kw.
7.	neoplasm$.ti,ab,kw.
8.	cancer$.ti,ab,kw.
9.	mesothelioma$.ti,ab,kw.
10.	1 or 2 or 3 or 4 or 5 or 6 or 7 or 8 or 9
11.	Electronic Nose/
12.	Volatile Organic Compounds/
13.	artificial nose$.ti,ab,kw.
14.	electronic nose$.ti,ab,kw.
15.	e-nose$.ti,ab,kw.
16.	eNose$.ti,ab,kw.
17.	VOC$.ti,ab,kw.
18.	volatile organic compound$.ti,ab,kw.
19.	breath anal$.ti,ab,kw.
20.	machine olfact$.ti,ab,kw.
21.	artificial olfact$.ti,ab,kw.
22.	aeonose$.ti,ab,kw.
23.	cyranose$.ti,ab,kw.
24.	11 or 12 or 13 or 14 or 15 or 16 or 17 or 18 or 19 or 20 or 21 or 22 or 23
25.	Diagnosis/
26.	"Sensitivity and Specificity"/
27.	diagnosis$.ti,ab,kw.
28.	diagnostic$.ti,ab,kw.
29.	sensitivity$.ti,ab,kw.
30.	specificity$.ti,ab,kw.
31.	ROC curve$.ti,ab,kw.
32.	ROC analys$.ti,ab,kw.
33.	receiver operating characteristic$.ti,ab,kw.
34.	25 or 26 or 27 or 28 or 29 or 30 or 31 or 32 or 33
35.	10 and 24 and 34
36.	remove duplicates from 35

© 2022 Scheepers MHMC et al. *JAMA Network Open.*

eTable 2: Modified QUADAS-2 assessment tool

		QUADAS-2 (modified)
RISK of BIAS	Patient selection	Were sampled patients representative of the intended population?
		Did the study include patients as well as healthy controls?
		Did the study avoid inappropriate exclusions?
		**Could the selection of patients have introduced bias?**
	Index test	Was the index test and interpretation of data performed in standardized and reproducible fashion?
		If a threshold was used, was it pre-specified?
		Validation of results performed (internal or external)?<br>Did they validate their results on a blinded sample?
		**Could the conduct or interpretation of the index test have introduced bias?**
	Reference standard	Is the reference standard likely to correctly classify the target condition?
		Were the reference standard results interpreted without knowledge of the results of the index test?
		Is the reference standard likely to correctly classify the control group?
		**Could the reference standard, its conduct, or its interpretation have introduced bias?**
	Flow and timing	Was there an appropriate interval between index test and reference standard?
		Did all patients receive a reference standard?
		Were index test and reference standard performed prior to therapeutic intervention?
		Did all patients receive the same reference standard?
		Were measures taken to limit exogenous influences such as food intake and smoking etc?
		Were all patients included in the analysis?
		**Could the patient flow and application of the tests have introduced bias?**
APPLICABILITY	Patient selection	Are there concerns that the included patients and setting do not match the review question?
	Index test	Are there concerns that the authors have not demonstrated suitable reproducibility and sensitivity of the chosen index test?
	Reference standard	Are there concerns that the target condition as defined by the reference standard does not match the question?

© 2022 Scheepers MHMC et al. *JAMA Network Open.*

eTable 3: The Rational Clinical Examination: Evidence-Based Clinical Diagnosis >TABLE: Levels of Evidence

Levels of Evidence[a]

Level of Evidence	Grade	Definition
1	A	Independent blinded comparison of sign or symptom results with a criterion standard of diagnosis among a large number of consecutive patients suspected of having the target condition
2	B	Independent blinded comparison of sign or symptom with a criterion standard of diagnosis among a small number of consecutive patients suspected of having the target condition
3	C	Independent blinded comparison of sign or symptom with a criterion standard of diagnosis among nonconsecutive patients suspected of having the target condition
4	C	Nonindependent comparison of sign or symptom with a criterion standard of diagnosis among samples of patients who obviously have the target condition plus, perhaps, normal individuals
5	C	Nonindependent comparison of sign or symptom with a standard of uncertain validity

[a]Modified from Holleman and Simel.

© 2022 Scheepers MHMC et al. *JAMA Network Open.*

eTable 4: Examples of reported confounding factors and measures to reduce influences.

Factor	Measure	References
**Food and beverages**	Fasting before breath sampling	(1-7, 9, 14, 22, 24-43)
**Smoking**	Cessation of smoking before breath sampling	(1, 2, 4, 6, 7, 9, 14, 24-26, 28, 30-33, 35, 36, 38, 40-45)
**Expiratory compartment**	Alveolar or mixed exhaled breath.	(1-8, 12, 14, 28, 30, 31, 34, 38, 41, 43, 45, 46)
**Sample collection**	Exhaled breath can be collected in breath sampling bags, Tenax tubes or directly exposed to electronic nose sensors.	Tedlar:<br>(1, 3, 5, 9, 25-27, 30, 31, 34, 36-40, 43, 45-47)<br><br>Mylar:<br>(1, 6-8, 12, 48)<br><br>Gassampler:<br>(2, 4, 14)<br><br>Other:<br>(22), (41)(16, 17)(29, 49)<br><br>Direct:<br>(11, 13, 15, 18-21, 23, 32, 33, 42, 44, 50, 51)
**Exercise**	Exercise may affect the composition of VOCs found in exhaled breath.<br><br>A period of resting before breath sampling might be beneficial.	Resting:<br>(22, 25, 33, 40, 43)
**Oral VOCs**	VOCs produced by the oral microbiome or lingering VOCs from diet might influence VOCs in exhaled breath.<br><br>Mouth rinsing	(22-27)
**Perfume aromatics**	Cessation of use of perfume, deodorant or pungent foods.	Perfume/aromatics/ mouth hygiene:<br>(3), (25, 28-33)

© 2022 Scheepers MHMC et al. *JAMA Network Open.*

1. Shehada N, Cancilla JC, Torrecilla JS, Pariente ES, Brönstrup G, Christiansen S, et al. Silicon Nanowire Sensors Enable Diagnosis of Patients via Exhaled Breath. ACS Nano. 2016;10(7):7047-57.
2. Amal H, Leja M, Funka K, Lasina I, Skapars R, Sivins A, et al. Breath testing as potential colorectal cancer screening tool. International Journal of Cancer. 2016;138(1):229-36.
3. Amal H, Shi DY, Ionescu R, Zhang W, Hua QL, Pan YY, et al. Assessment of ovarian cancer conditions from exhaled breath. Int J Cancer. 2015;136(6):E614-22.
4. Amal H, Leja M, Funka K, Skapars R, Sivins A, Ancans G, et al. Detection of precancerous gastric lesions and gastric cancer through exhaled breath. Gut. 2016;65(3):400-7.
5. Barash O, Zhang W, Halpern JM, Hua QL, Pan YY, Kayal H, et al. Differentiation between genetic mutations of breast cancer by breath volatolomics. Oncotarget. 2015;6(42):44864-76.
6. Broza YY, Kremer R, Tisch U, Gevorkyan A, Shiban A, Best LA, et al. A nanomaterial-based breath test for short-term follow-up after lung tumor resection. Nanomedicine: Nanotechnology, Biology and Medicine. 2013;9(1):15-21.
7. Gruber M, Tisch U, Jeries R, Amal H, Hakim M, Ronen O, et al. Analysis of exhaled breath for diagnosing head and neck squamous cell carcinoma: a feasibility study. British Journal of Cancer. 2014;111(4):790-8.
8. Hakim M, Billan S, Tisch U, Peng G, Dvrokind I, Marom O, et al. Diagnosis of head-and-neck cancer from exhaled breath. British Journal of Cancer. 2011;104(10):1649-55.
9. Hubers AJ, Brinkman P, Boksem RJ, Rhodius RJ, Witte BI, Zwinderman AH, et al. Combined sputum hypermethylation and eNose analysis for lung cancer diagnosis. J Clin Pathol. 2014;67(8):707-11.
10. Kort S, Tiggeloven MM, Brusse-Keizer M, Gerritsen JW, Schouwink JH, Citgez E, et al. Multi-centre prospective study on diagnosing subtypes of lung cancer by exhaled-breath analysis. Lung Cancer. 2018;125:223-9.
11. Kort S, Brusse-Keizer M, Gerritsen JW, Schouwink H, Citgez E, de Jongh F, et al. Improving lung cancer diagnosis by combining exhaled-breath data and clinical parameters. ERJ Open Res. 2020;6(1):00221-2019.
12. Peled N, Hakim M, Bunn PA, Jr., Miller YE, Kennedy TC, Mattei J, et al. Non-invasive breath analysis of pulmonary nodules. J Thorac Oncol. 2012;7(10):1528-33.
13. Schuermans VNE, Li Z, Jongen ACHM, Wu Z, Shi J, Ji J, et al. Pilot Study: Detection of Gastric Cancer From Exhaled Air Analyzed With an Electronic Nose in Chinese Patients. Surg Innov. 2018;25(5):429-34.
14. Shlomi D, Abud M, Liran O, Bar J, Gai-Mor N, Ilouze M, et al. Detection of Lung Cancer and EGFR Mutation by Electronic Nose System. J Thorac Oncol. 2017;12(10):1544-51.
15. Steenhuis EGM, Schoenaker IJH, de Groot JWB, Fiebrich HB, de Graaf JC, Brohet RM, et al. Feasibility of volatile organic compound in breath analysis in the follow-up of colorectal cancer: A pilot study. Eur J Surg Oncol. 2020.
16. Tirzite M, Bukovskis M, Strazda G, Jurka N, Taivans I. Detection of lung cancer in exhaled breath with an electronic nose using support vector machine analysis. Journal of breath research. 2017;11(3):036009.
17. Tirzite M, Bukovskis M, Strazda G, Jurka N, Taivans I. Ao - Tirzite M, http://orcid.org O. Detection of lung cancer with electronic nose and logistic regression analysis. Journal of Breath Research. 2019;13(1):016006.
18. van de Goor RMGE, van Hooren MRA, Henatsch D, Kremer B, Kross K.W. Ao - van de Goor RMGE, Orcid: http://orcid.org/--- Ao - Kross KW, et al. Detecting head and neck squamous carcinoma using a portable handheld electronic nose. Head Neck. 2020;42(9):2555-9.
19. van de Goor R, van Hooren M, Dingemans AM, Kremer B, Kross K. Ao - van de Goor R, http://orcid.org O. Training and Validating a Portable Electronic Nose for Lung Cancer Screening. J Thorac Oncol. 2018;13(5):676-81.
20. Mohamed N, van de Goor R, El-Sheikh M, Elrayah O, Osman T, Nginamau ES, et al. Feasibility of a Portable Electronic Nose for Detection of Oral Squamous Cell Carcinoma in Sudan. Healthcare. 2021;9(5):534.
21. Waltman CG, Marcelissen TAT, van Roermund JGH. Exhaled-breath Testing for Prostate Cancer Based on Volatile Organic Compound Profiling Using an Electronic Nose Device (Aeonose™): A Preliminary Report. Eur Urol Focus. 2020;6(6):1220-5.
22. Chapman EA, Thomas PS, Stone E, Lewis C, Yates DH. A breath test for malignant mesothelioma using an electronic nose. Eur Respir J. 2012;40(2):448-54.
23. de Vries R, Brinkman P, van der Schee MP, Fens N, Dijkers E, Bootsma SK, et al. Integration of electronic nose technology with spirometry: validation of a new approach for exhaled breath analysis. J Breath Res. 2015;9(4):046001.
24. Kononov A, Korotetsky B, Jahatspanian I, Gubal A, Vasiliev A, Arsenjev A, et al. Online breath analysis using metal oxide semiconductor sensors (electronic nose) for diagnosis of lung cancer. J Breath Res. 2019;14(1):016004.

© 2022 Scheepers MHMC et al. *JAMA Network Open.*

25. Li W, Jia Z, Xie D, Chen K, Cui J, Liu H. Recognizing lung cancer using a homemade e-nose: A comprehensive study. Comput Biol Med. 2020;120:103706.
26. Liu L, Li W, He Z, Chen W, Liu H, Chen K, et al. Detection of lung cancer with electronic nose using a novel ensemble learning framework. J Breath Res. 2021.
27. Chen K, Liu L, Nie B, Lu B, Fu L, He Z, et al. Recognizing lung cancer and stages using a self-developed electronic nose system. Comput Biol Med. 2021;131:104294.
28. Diaz de Leon-Martinez L, Rodriguez-Aguilar M, Gorocica-Rosete P, Dominguez Reyes CA, Martinez Bustos V, Tenorio-Torres JA, et al. Identification of profiles of volatile organic compounds in exhaled breath by means of an electronic nose as a proposal for a screening method for breast cancer: a case-control study. Journal of breath research. 2020.
29. Di Natale C, Macagnano A, Martinelli E, Paolesse R, D'Arcangelo G, Roscioni C, et al. Lung cancer identification by the analysis of breath by means of an array of non-selective gas sensors. Biosensors and Bioelectronics. 2003;18(10):1209-18.
30. Gasparri R, Santonico M, Valentini C, Sedda G, Borri A, Petrella F, et al. Volatile signature for the early diagnosis of lung cancer. J Breath Res. 2016;10(1):016007.
31. Marzorati D, Mainardi L, Sedda G, Gasparri R, Spaggiari L, Cerveri P, editors. A Metal Oxide Gas Sensors Array for Lung Cancer Diagnosis Through Exhaled Breath Analysis. 2019 41st Annual International Conference of the IEEE Engineering in Medicine and Biology Society (EMBC); 2019 23-27 July 2019.
32. Rocco R, Incalzi RA, Pennazza G, Santonico M, Pedone C, Bartoli IR, et al. BIONOTE e-nose technology may reduce false positives in lung cancer screening programmes†. Eur J Cardiothorac Surg. 2016;49(4):1112-7; discussion 7.
33. Leja M, Kortelainen JM, Polaka I, Turppa E, Mitrovics J, Padilla M, et al. Sensing gastric cancer via point-of-care sensor breath analyzer. Cancer. 2021;127(8):1286-92.
34. Altomare DF, Porcelli F, Picciariello A, Pinto M, Di Lena M, Caputi Iambrenghi O, et al. The use of the PEN3 e-nose in the screening of colorectal cancer and polyps. Tech Coloproctol. 2016;20(6):405-9.
35. Chen Q, Chen Z, Liu D, He Z, Wu J. Constructing an E-Nose Using Metal-Ion-Induced Assembly of Graphene Oxide for Diagnosis of Lung Cancer via Exhaled Breath. ACS Applied Materials & Interfaces. 2020;12(15):17713-24.
36. Dragonieri S, Annema JT, Schot R, van der Schee MPC, Spanevello A, Carratu P, et al. An electronic nose in the discrimination of patients with non-small cell lung cancer and COPD. Lung Cancer. 2009;64(2):166-70.
37. Dragonieri S, van der Schee MP, Massaro T, Schiavulli N, Brinkman P, Pinca A, et al. An electronic nose distinguishes exhaled breath of patients with Malignant Pleural Mesothelioma from controls. Lung Cancer. 2012;75(3):326-31.
38. Huang C-H, Zeng C, Wang Y-C, Peng H-Y, Lin C-S, Chang C-J, et al. A Study of Diagnostic Accuracy Using a Chemical Sensor Array and a Machine Learning Technique to Detect Lung Cancer. Sensors (Basel). 2018;18(9).
39. Leunis N, Boumans M-L, Kremer B, Din S, Stobberingh E, Kessels AGH, et al. Application of an electronic nose in the diagnosis of head and neck cancer. Laryngoscope. 2014;124(6):1377-81.
40. Li W, Liu H, Xie D, He Z, Pi X. Lung Cancer Screening Based on Type-different Sensor Arrays. Scientific Reports. 2017;7(1):1969.
41. Raspagliesi F, Bogani G, Benedetti S, Grassi S, Ferla S, Buratti S. Detection of ovarian cancer through exhaled breath by electronic nose: A prospective study. Cancers. 2020;12(9):1-13.
42. Tan J-L, Yong Z-X, Liam C-K. Using a chemiresistor-based alkane sensor to distinguish exhaled breaths of lung cancer patients from subjects with no lung cancer. J Thorac Dis. 2016;8(10):2772-83.
43. Xu Zq, Broza YY, Ionsecu R, Tisch U, Ding L, Liu H, et al. A nanomaterial-based breath test for distinguishing gastric cancer from benign gastric conditions. British Journal of Cancer. 2013;108(4):941-50.
44. Krauss E, Haberer J, Barreto G, Degen M, Seeger W, Guenther A. Ao - Krauss E, et al. Recognition of breathprints of lung cancer and chronic obstructive pulmonary disease using the Aeonoseelectronic nose. Journal of Breath Research. 2020;14(4):046004.
45. Yang H-Y, Wang Y-C, Peng H-Y, Huang C-H. Breath biopsy of breast cancer using sensor array signals and machine learning analysis. Scientific Reports. 2021;11(1):103.
46. Capuano R, Santonico M, Pennazza G, Ghezzi S, Martinelli E, Roscioni C, et al. The lung cancer breath signature: a comparative analysis of exhaled breath and air sampled from inside the lungs. Scientific Reports. 2015;5(1):16491.
47. Lamote K, Brinkman P, Vandermeersch L, Vynck M, Sterk PJ, Van Langenhove H, et al. Breath analysis by gas chromatography-mass spectrometry and electronic nose to screen for pleural mesothelioma: A crosssectional case-control study. Oncotarget. 2017;8(53):91593-602.
48. Machado RF, Laskowski D, Deffenderfer O, Burch T, Zheng S, Mazzone PJ, et al. Detection of lung cancer by sensor array analyses of exhaled breath. American Journal of Respiratory and

Critical Care Medicine. 2005;171(11):1286-91.
49. Mohamed EI, Mohamed MA, Abdel-Mageed SM, Abdel-Mohdy TS, Badawi MI, Darwish SH. Volatile organic compounds of biofluids for detecting lung cancer by an electronic nose based on artificial neural network. J App Biomed. 2019;17(1):61-7.
50. van de Goor RMGE, Hardy JCA, van Hooren MRA, Kremer B, Kross K.W. Ao - van de Goor RMGE, http://orcid.org O. Detecting recurrent head and neck cancer using electronic nose technology: A feasibility study. Head Neck. 2019;41(9):2983-90.
51. van Keulen KE, Jansen ME, Schrauwen RWM, Kolkman JJ, Siersema PD. Volatile organic compounds in breath can serve as a non-invasive diagnostic biomarker for the detection of advanced adenomas and colorectal cancer. Aliment Pharmacol Ther. 2020;51(3):334-46.
52. Herman-Saffar O, Boger Z, Libson S, Lieberman D, Gonen R, Zeiri Y. Early non-invasive detection of breast cancer using exhaled breath and urine analysis. Comput Biol Med. 2018;96:227-32.

© 2022 Scheepers MHMC et al. *JAMA Network Open.*

eTable 5: Quality assessment using QUADAS-2 tool: Summary and separate outcome of risk of bias and concerns regarding applicability for included studies.

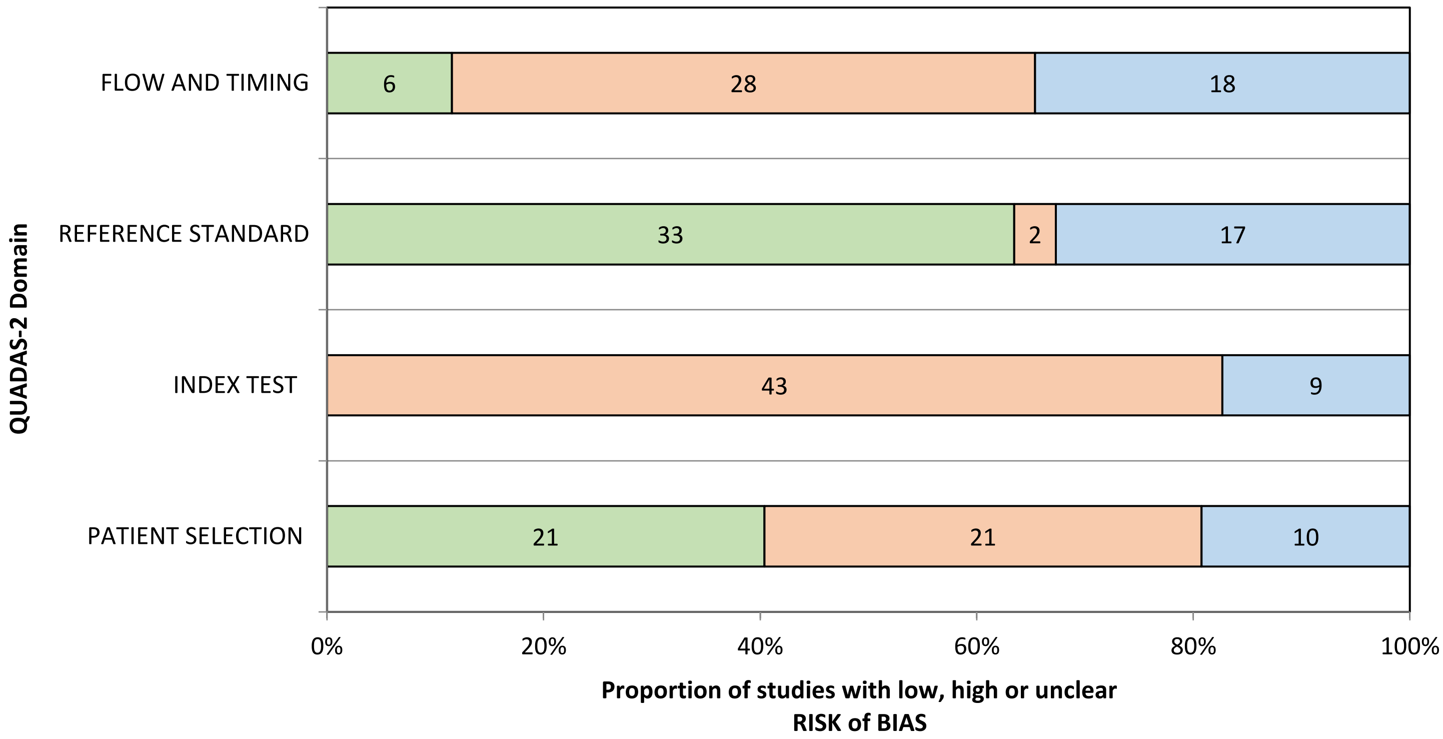

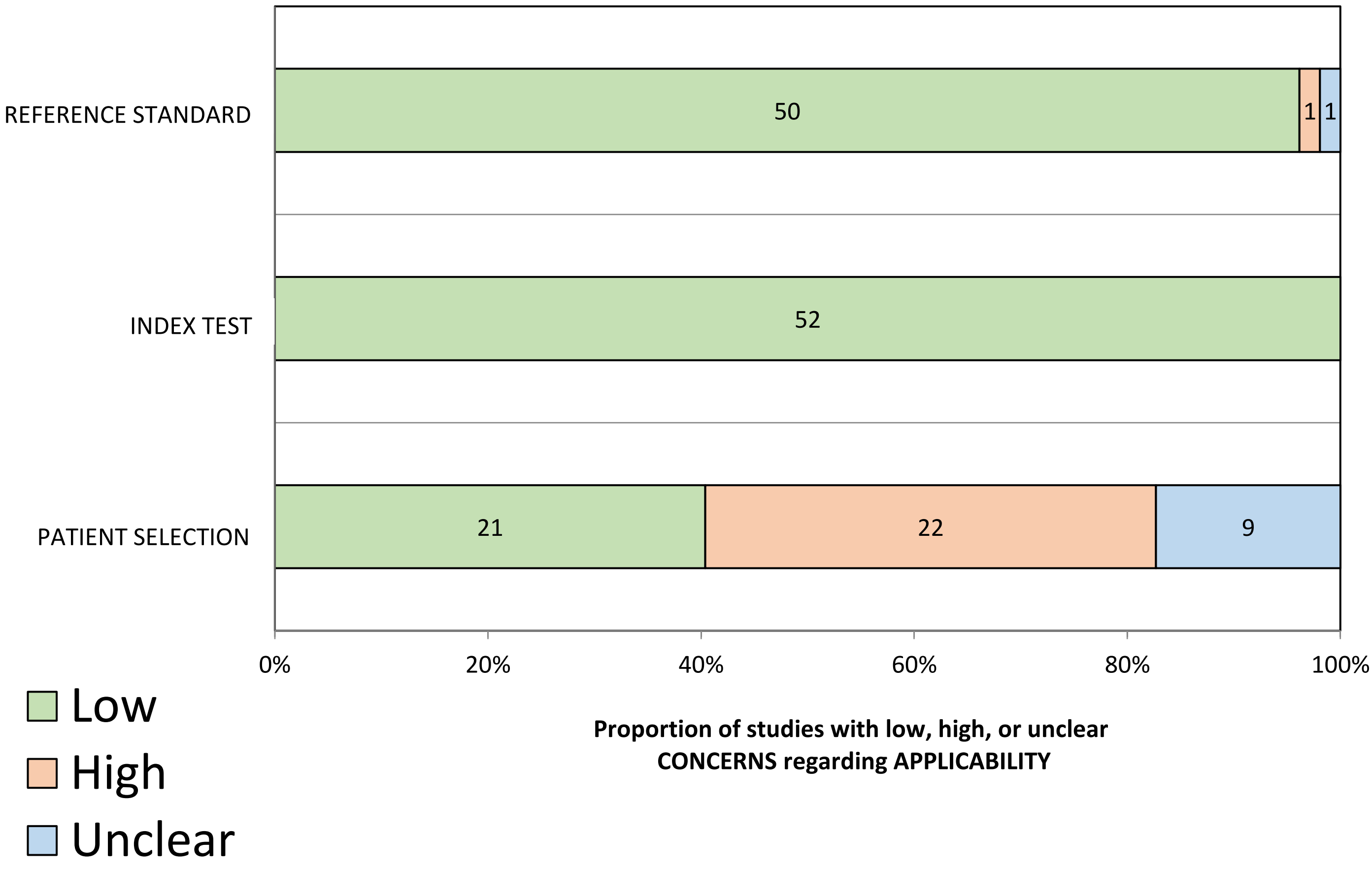

© 2022 Scheepers MHMC et al. *JAMA Network Open.*

eTable 6: Quality assessment using QUADAS-2 tool: Outcomes of risk of bias and applicability concerns assessment for individual studies

Study	Risk of Bias				Applicability concerns		
	Patient Selection	Index test	Reference standard	Flow and timing	Patient selection	Index test	Reference standard
Altomare et al, 41 2016	☺	☹	☺	?	☺	☺	☺
Amal et al,6 2014	☹	☹	☺	?	?	☺	☺
Amal et al, 44 2015	?	?	☺	?	?	☺	☺
Amal et al, 45 2016	☺	?	☺	?	☺	☺	☺
Barash et al,64 2015	☺	?	☺	?	☺	☺	☺
Broza et al, 46 2012	☺	☹	☺	☹	☺	☺	☺
Capuano et al, 65 2015	☹	☹	?	☹	☹	☺	☺
Chapman et al, 32 2012	?	☹	☺	☹	☺	☺	☺
Chen et al, 47 2021	☹	?	☹	☹	☹	☺	?
Chen Q et al, 48 2020	☺	☹	☺	?	☺	☺	☺
de Kort et al, 23 2018	☺	☹	?	☹	☹	☺	☺
de Kort et al, 22 2020	☺	☹	☺	☹	☹	☺	☺
de Vries et al,66 2015	☹	☹	☺	☹	☹	☺	☺
Di Natale et al,67 2003	☹	☹	?	☹	☹	☺	☺
Diaz de Leon-Martinez et al, 33 2020	☺	☹	☺	?	☺	☺	☺
Dragonieri et al, 68 2009	☹	☹	☺	?	☹	☺	☺
Dragonieri et al, 16 2012	☹	☹	☺	☹	☹	☺	☺
Gasparri et al, 49 2016	☹	☹	☺	☹	☹	☺	☺

© 2022 Scheepers MHMC et al. *JAMA Network Open.*

Gruber et al, 50 2014	☺	?	☺	☺	☹	☺	☺
Hakim et al,51 2011	?	☹	?	☹	☺	☺	☺
Herman-Saffar et al, 34 2018	☹	☹	?	☹	?	☺	☺
Huang et al, 35 2018	☹	☹	☺	☺	☹	☺	☺
Hubers et al, 36 2014	?	☹	☺	☹	?	☺	☺
Kononov et al, 52 2019	☺	☹	?	?	☹	☺	☺
Krauss et al, 24 2020	?	☹	☺	☹	☹	☺	☺
Lamote et al, 37 2017	☺	☹	?	?	☺	☺	☺
Leja et al,53 2021	☺	?	☺	?	☹	☺	☺
Leunis et al, 54 2014	☹	☹	?	☹	☺	☺	☺
Li et al, 55 2017	☹	?	?	☹	☺	☺	☺
Li et al, 69 2020	☹	?	☺	☹	☹	☺	☺
Liu et al, 56 2021	☺	☹	☺	☹	☺	☺	☺
Machado et al, 17 2005	☺	☹	☺	☹	☹	☺	☺
Marzorati et al, 57 2019	☹	☹	?	☹	?	☺	☺
Mohamed et al, 25 2021	?	☹	☺	☹	?	☺	☺
Mohamed et al, 42 2019	☹	☹	☺	☺	☺	☺	☺
Peled et al,58 2012	☺	☹	☺	☹	☺	☺	☺
Raspagliesi et al, 43 2020	☺	☹	☺	☺	☹	☺	☺
Rocco et al, 59 2016	?	☹	?	?	?	☺	☺
Schuermans et al, 26 2018	☺	☹	☺	?	?	☺	☺

© 2022 Scheepers MHMC et al. *JAMA Network Open.*

Shehada et al, 60 2016	☹	☹	?	☹	☹	☺	☺
Shlomi et al, 61 2017	☺	☹	☺	☺	☺	☺	☺
Steenhuis et al, 27 2020	☺	☹	☺	?	☺	☺	☺
Tan et al, 62 2016	☺	☹	☺	☹	☺	☺	☺
Tirzite et al,38 2017	?	☹	?	?	☹	☺	☺
Tirzite et al, 39 2019	?	☹	?	?	☹	☺	☺
van de Goor et al, 28 2018	☹	☹	?	☹	☹	☺	☺
van de Goor et al, 18 2019	☺	☹	?	?	☺	☺	☺
van de Goor et al, 29 2020	☹	☹	?	☹	☹	☺	☺
van Keulen et al, 30 2020	?	☹	☺	?	☺	☺	☺
Waltman, 31 2020	☹	☹	☺	☹	?	☺	☺
Xu et al,63 2013	☹	?	☺	☺	☺	☺	☺
Yang et al, 40 2021	☹	☹	☹	☹	☺	☺	☹

☺ low risk ☹ high risk ? unclear risk

© 2022 Scheepers MHMC et al. *JAMA Network Open.*

eFigure 1: Outlier and Influence analysis of all e-nose studies.

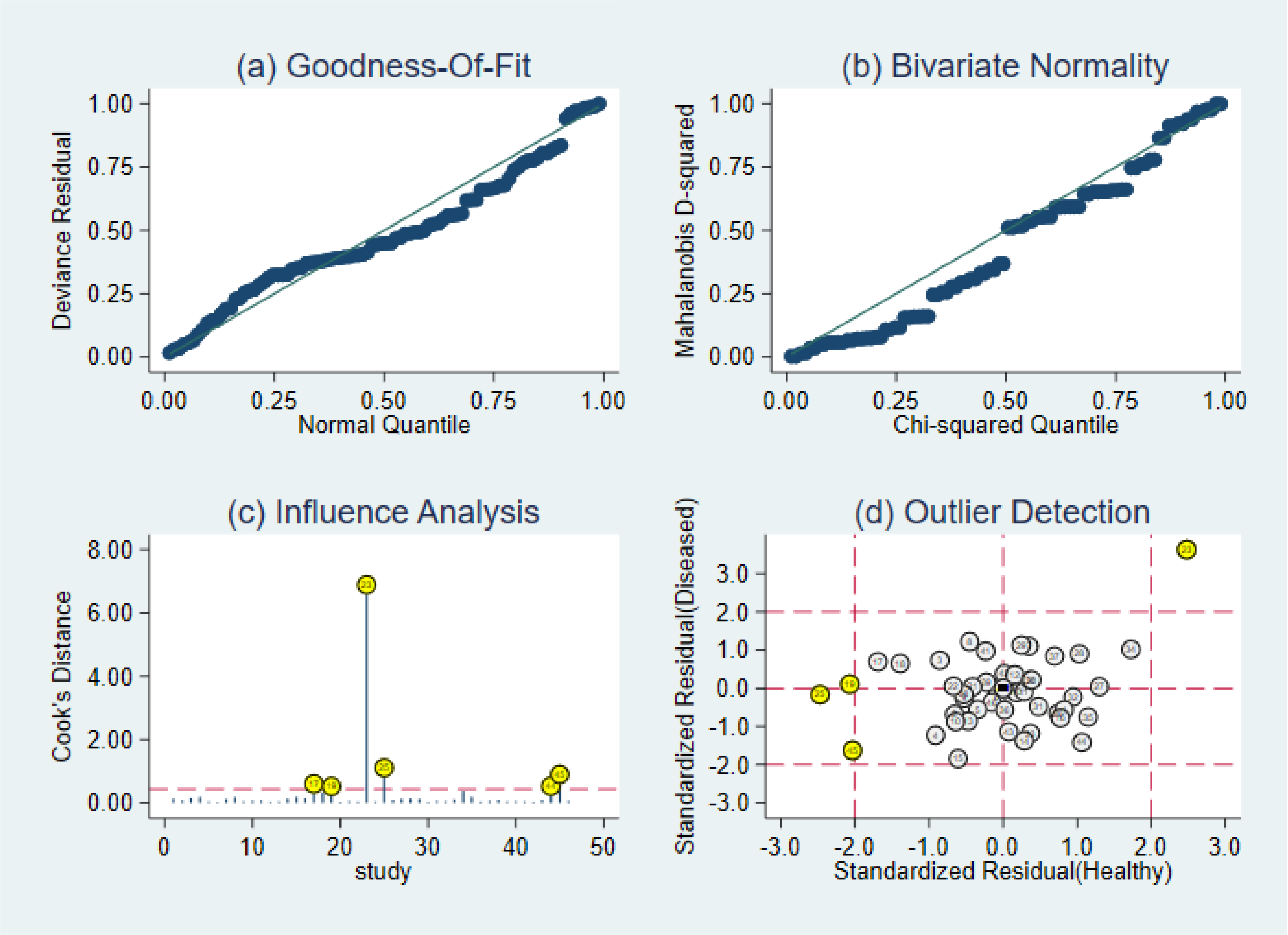

© 2022 Scheepers MHMC et al. *JAMA Network Open.*

eFigure 2: Pooled analysis of all e-nose studies after exclusion of outliers

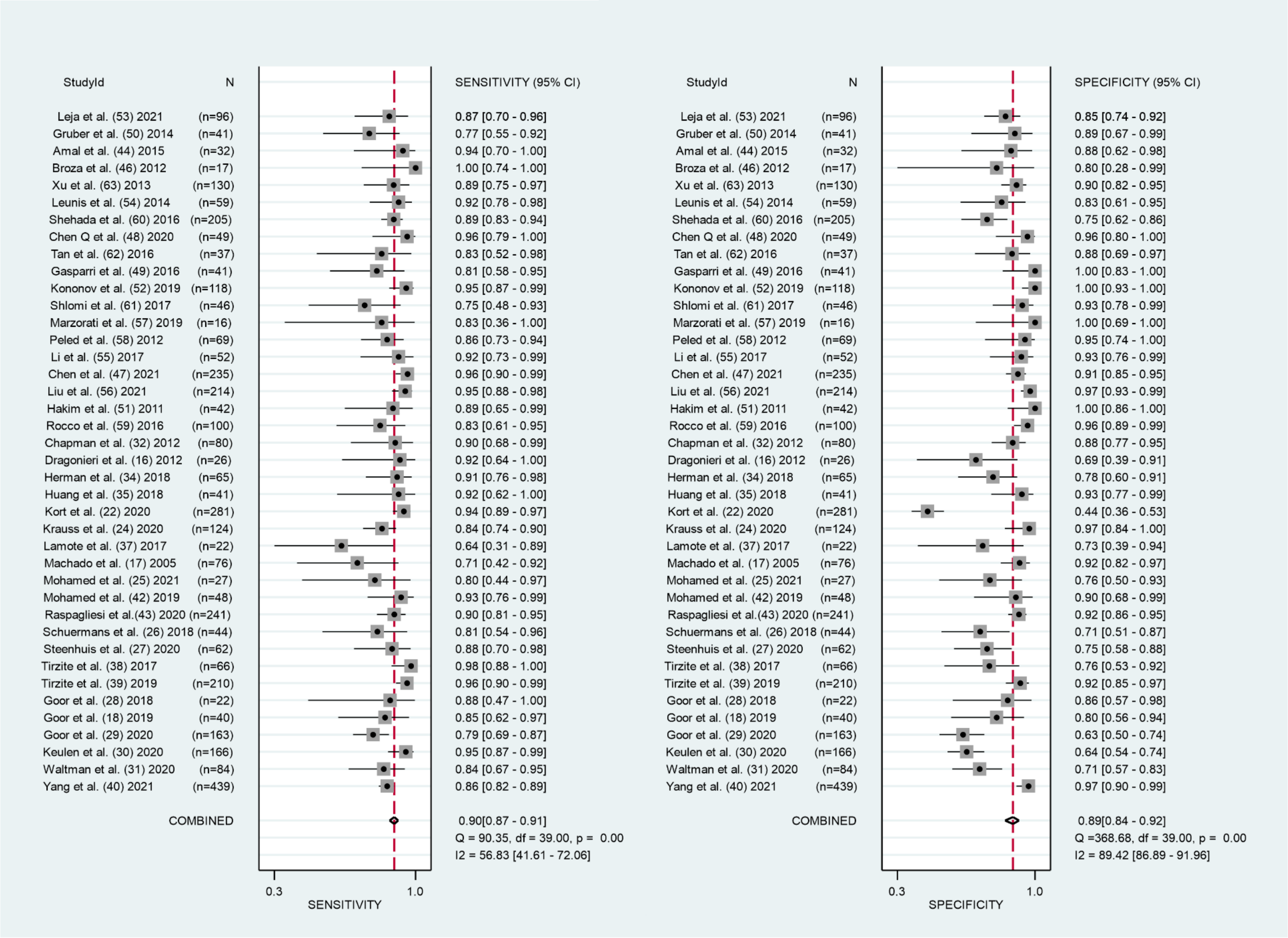

© 2022 Scheepers MHMC et al. *JAMA Network Open.*

eFigure 3: Publication bias analysis for all e-nose studies.

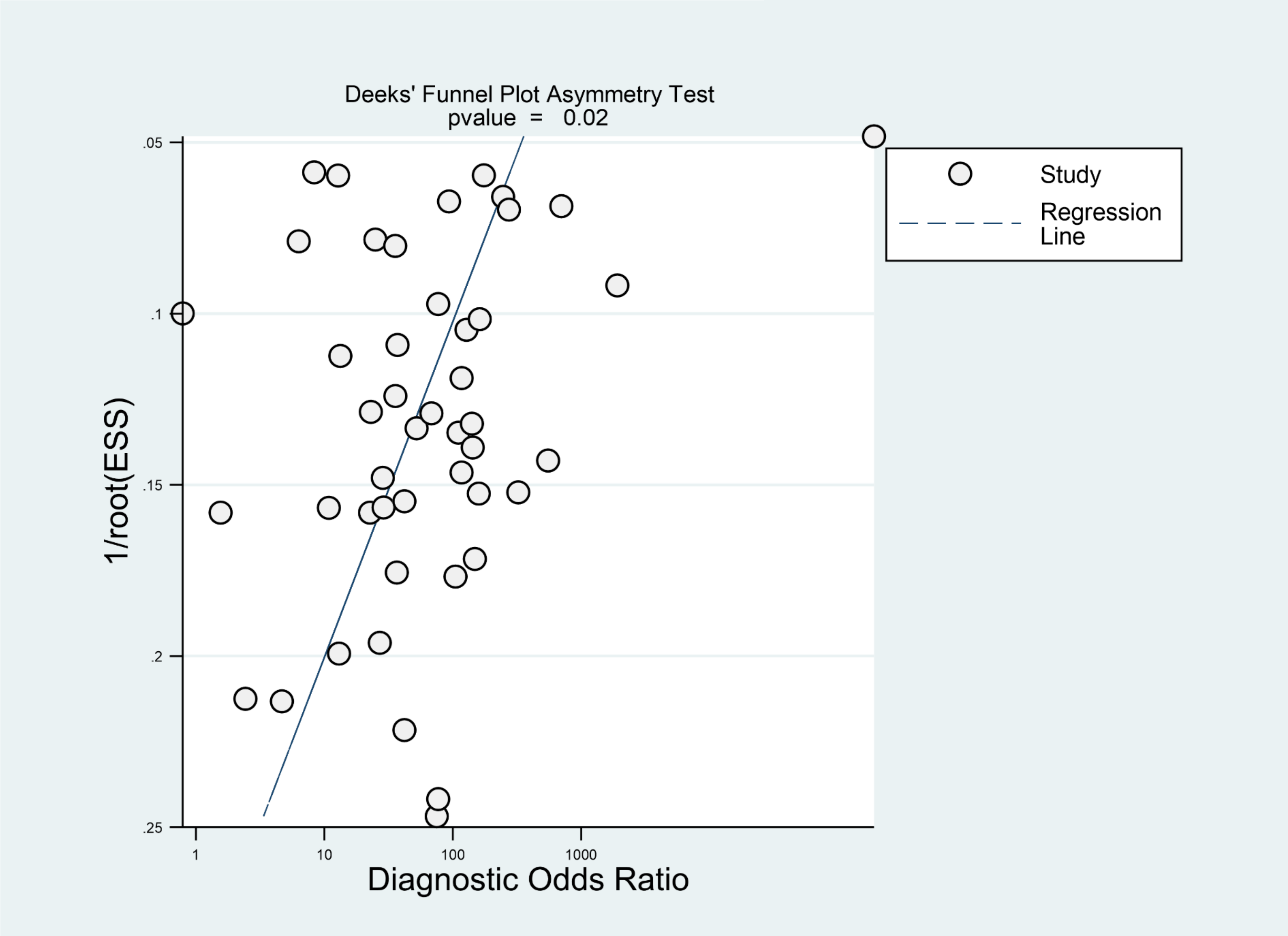

© 2022 Scheepers MHMC et al. *JAMA Network Open.*

eFigure 4: Pooled analysis of all Cyranose320 studies

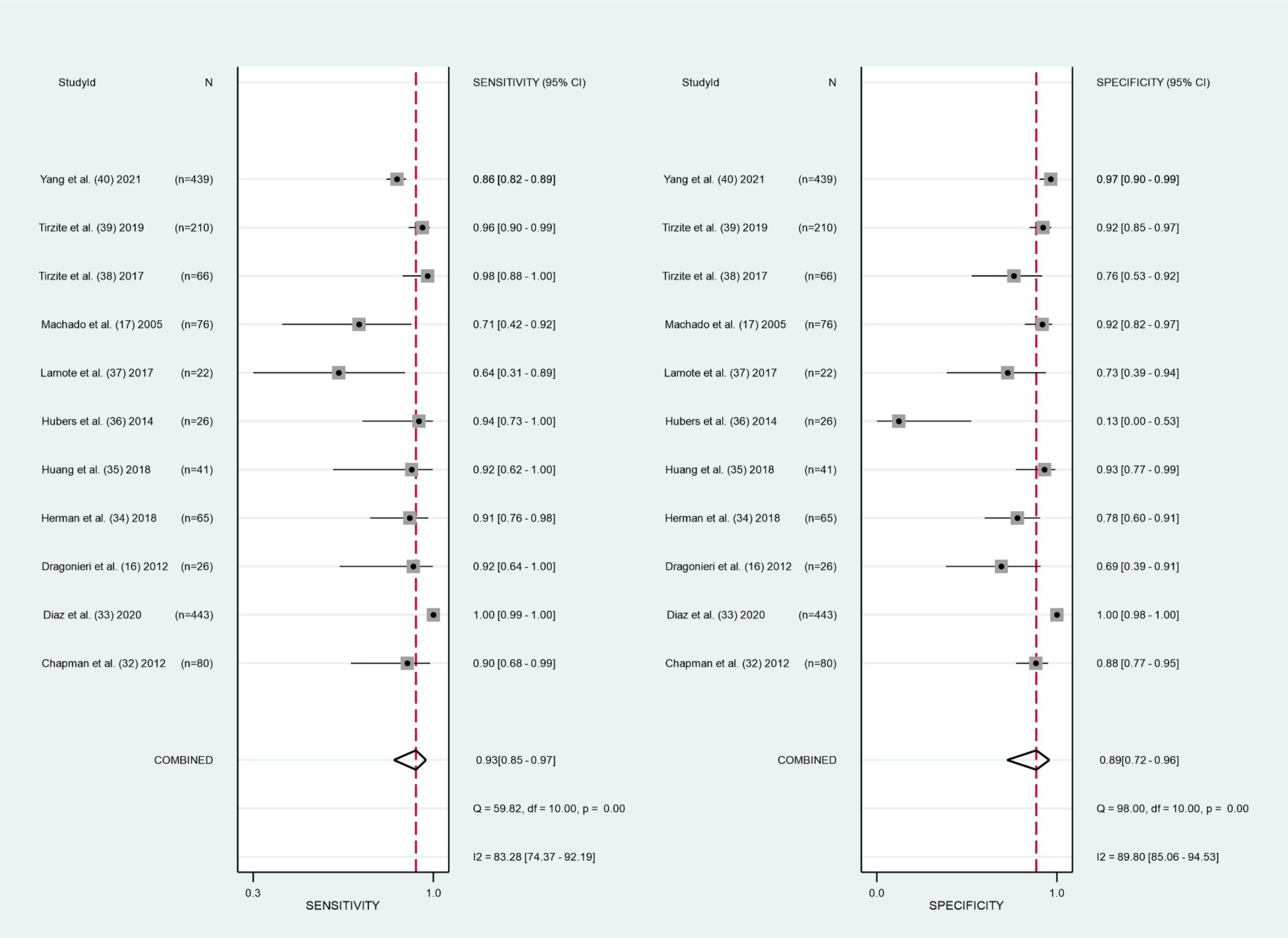

© 2022 Scheepers MHMC et al. *JAMA Network Open.*

eFigure 5: Pooled analysis of all Aeonose studies

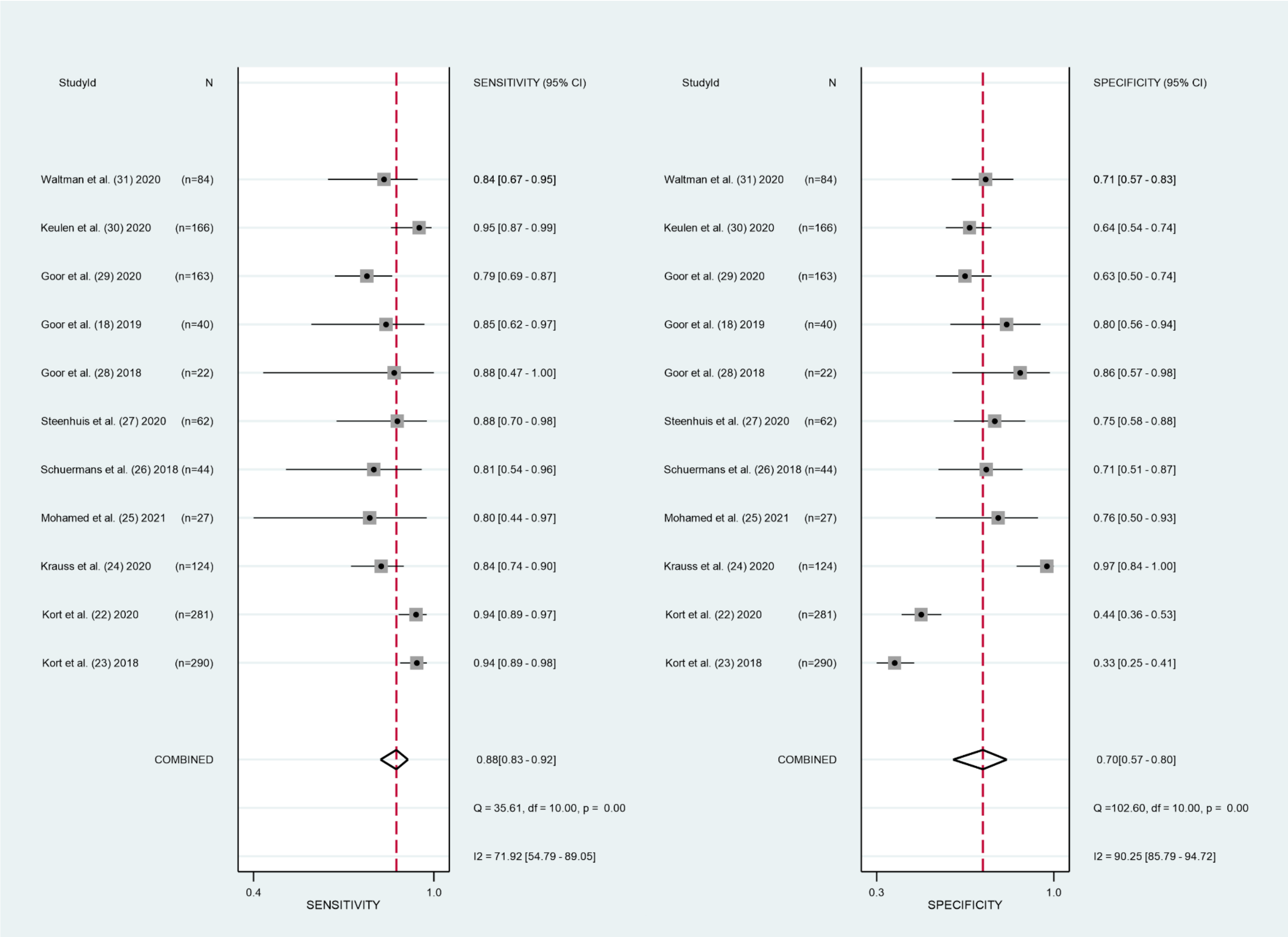

© 2022 Scheepers MHMC et al. *JAMA Network Open.*

eFigure 6: Pooled analysis of all lung cancer studies

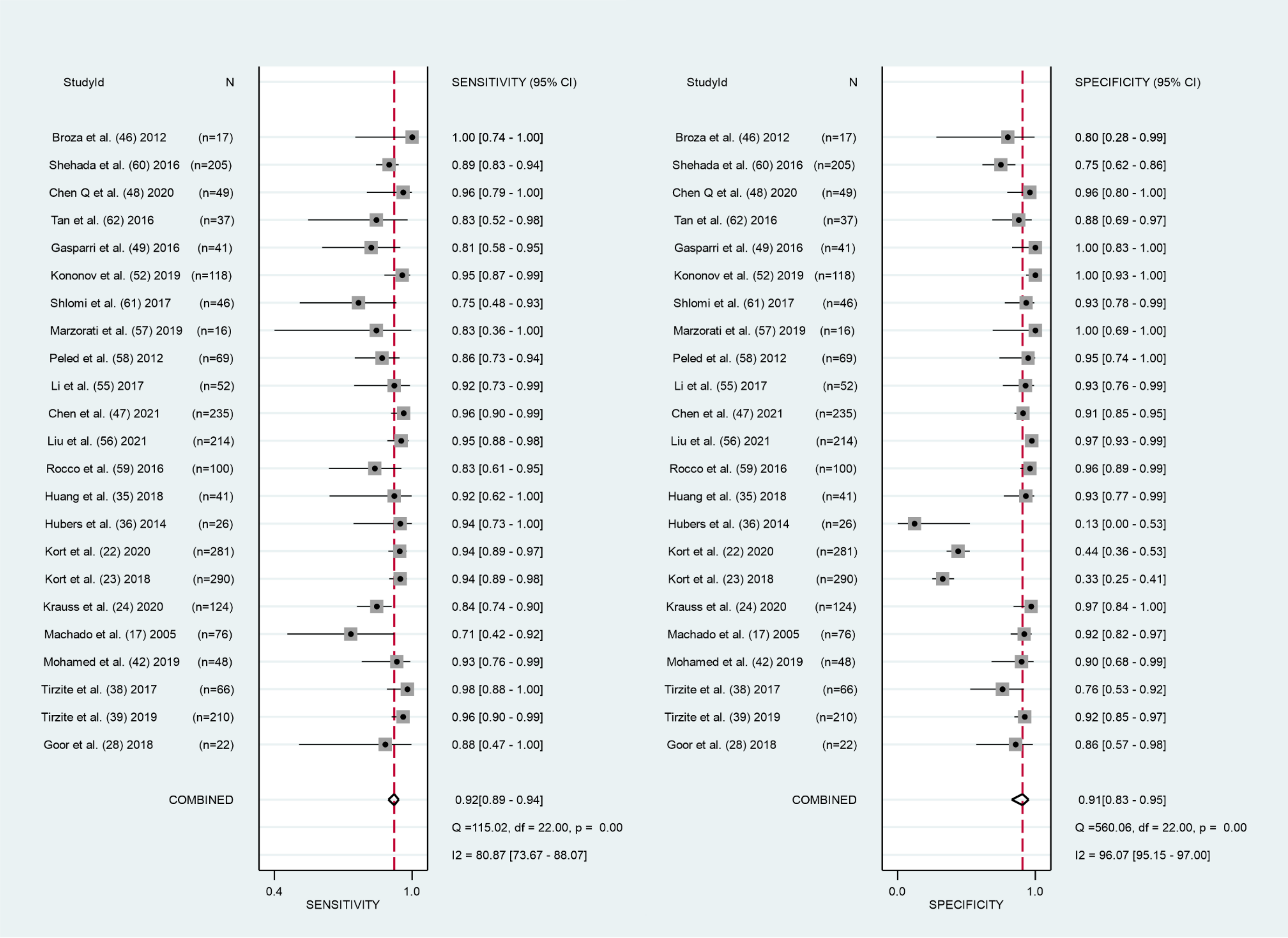

© 2022 Scheepers MHMC et al. *JAMA Network Open.*

eFigure 7: Pooled analysis of all head and neck cancer studies

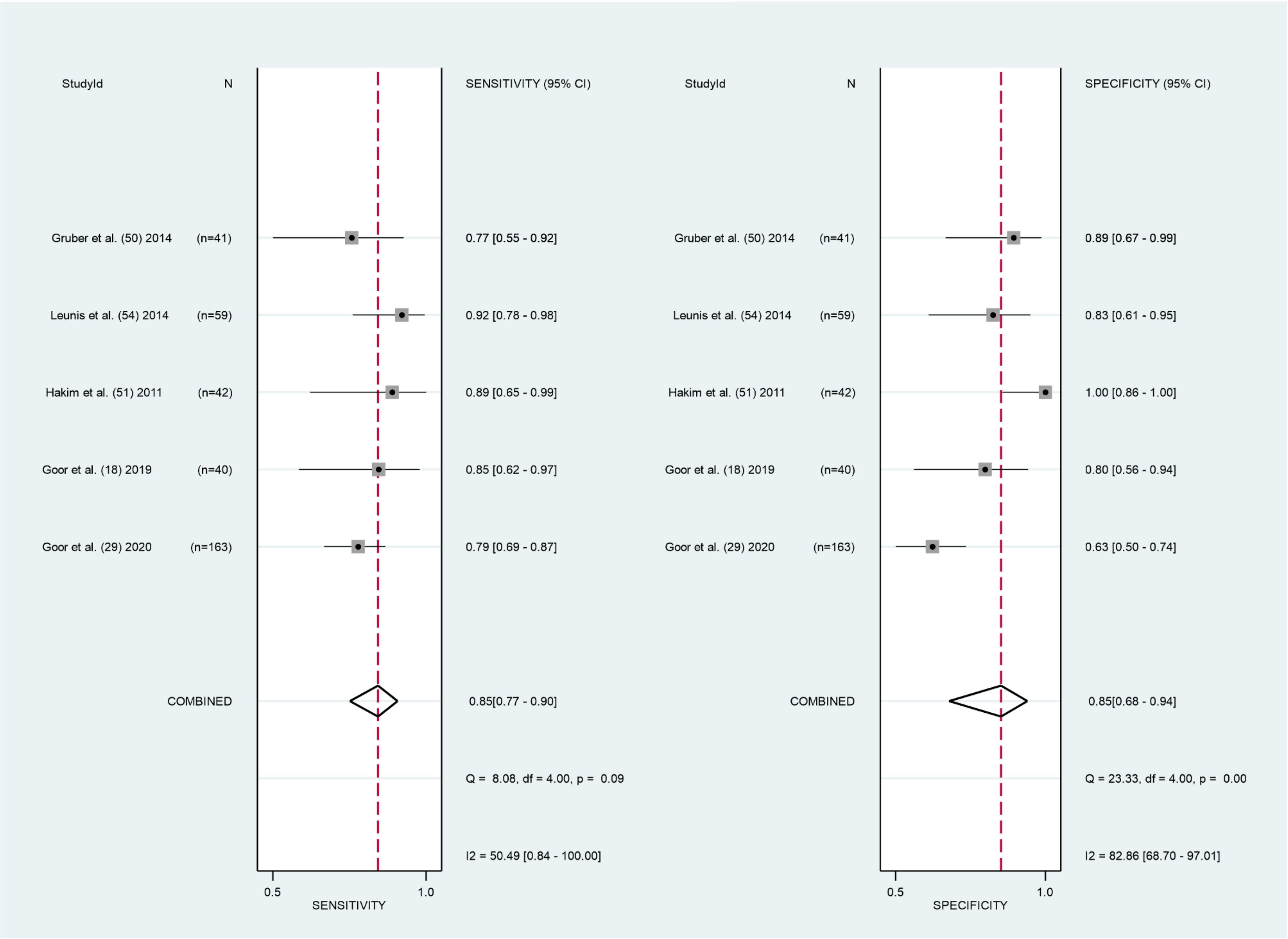

© 2022 Scheepers MHMC et al. *JAMA Network Open.*

eFigure 8: Pooled analysis of all colorectal cancer studies

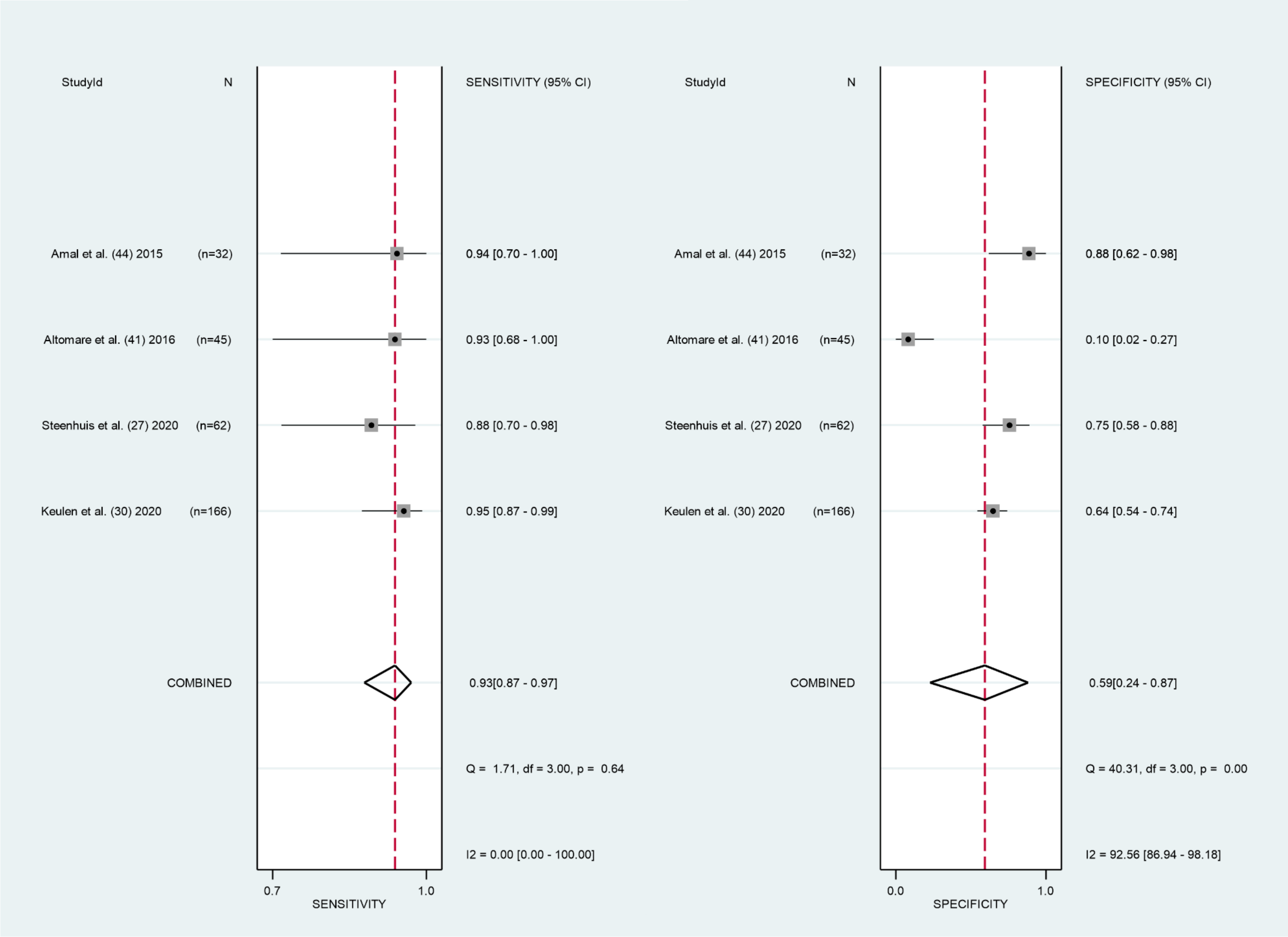

© 2022 Scheepers MHMC et al. *JAMA Network Open.*

eFigure 9: Pooled analysis of all advanced cancer stage studies

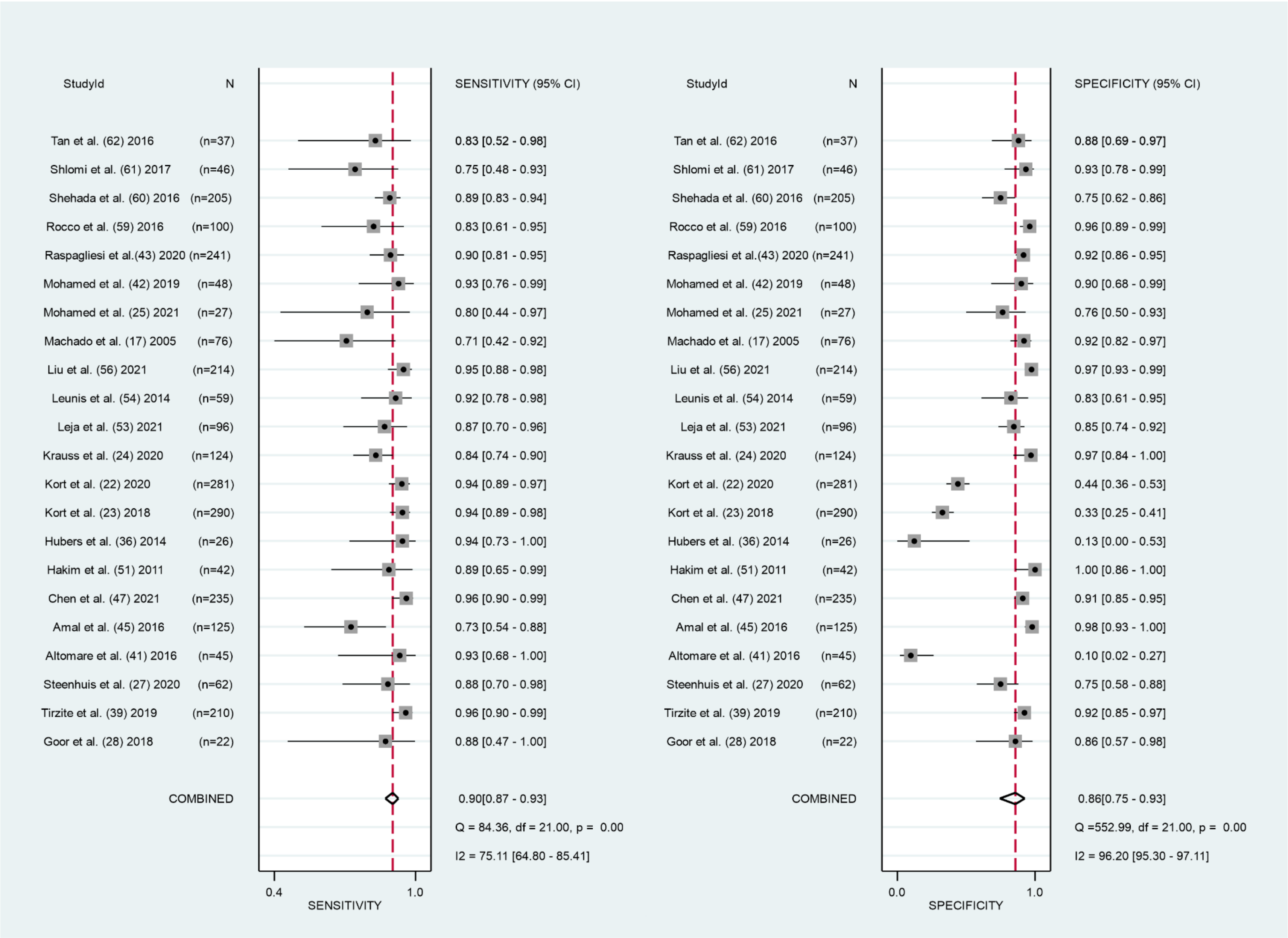

© 2022 Scheepers MHMC et al. *JAMA Network Open.*

eFigure 10: Pooled analysis of all early cancer stage studies

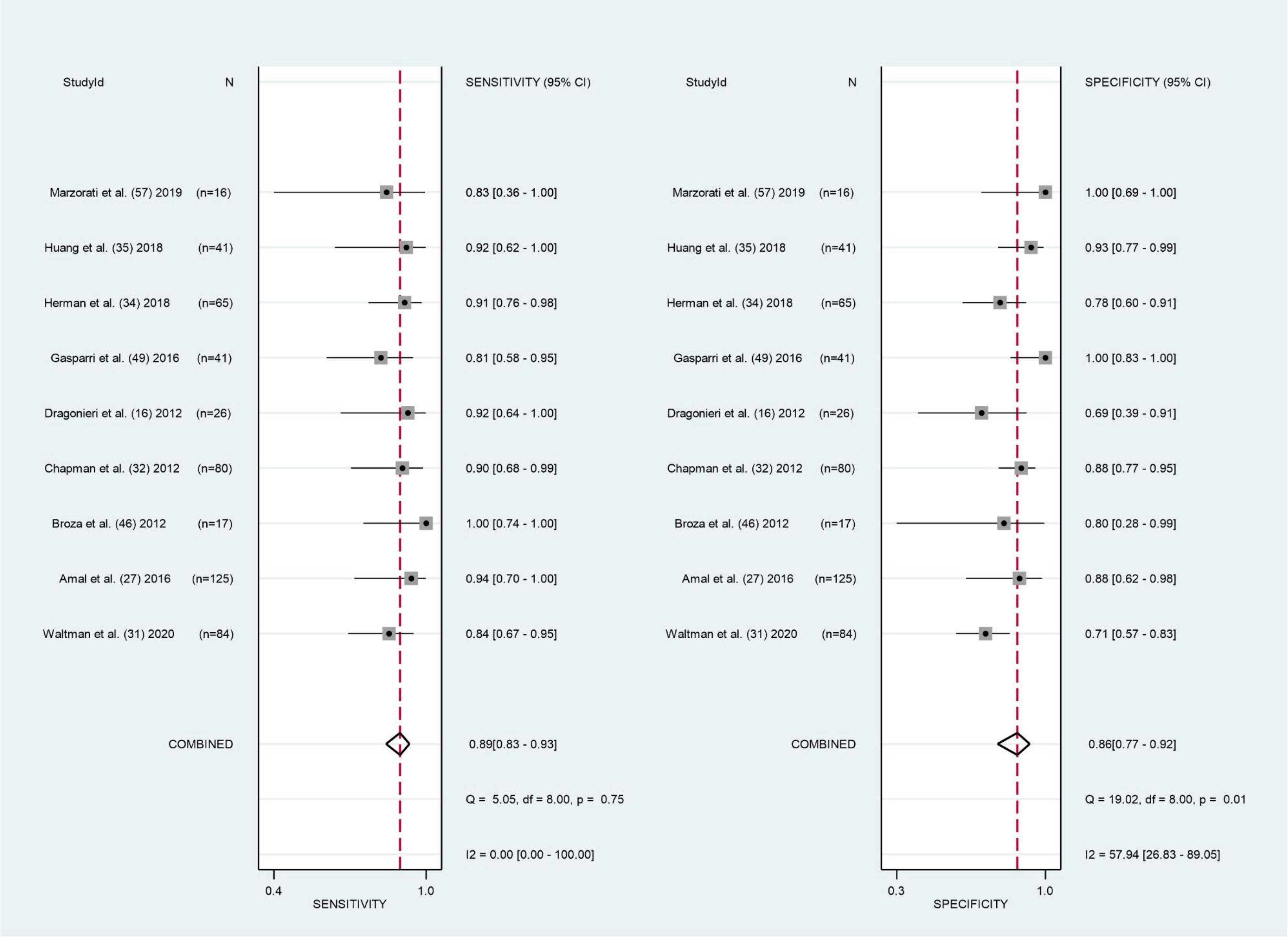

© 2022 Scheepers MHMC et al. *JAMA Network Open.*

eFigure 11: Pooled analysis of all studies after exclusion of studies with a high risk of bias on the Patient Selection domain of the QUADAS-2 tool

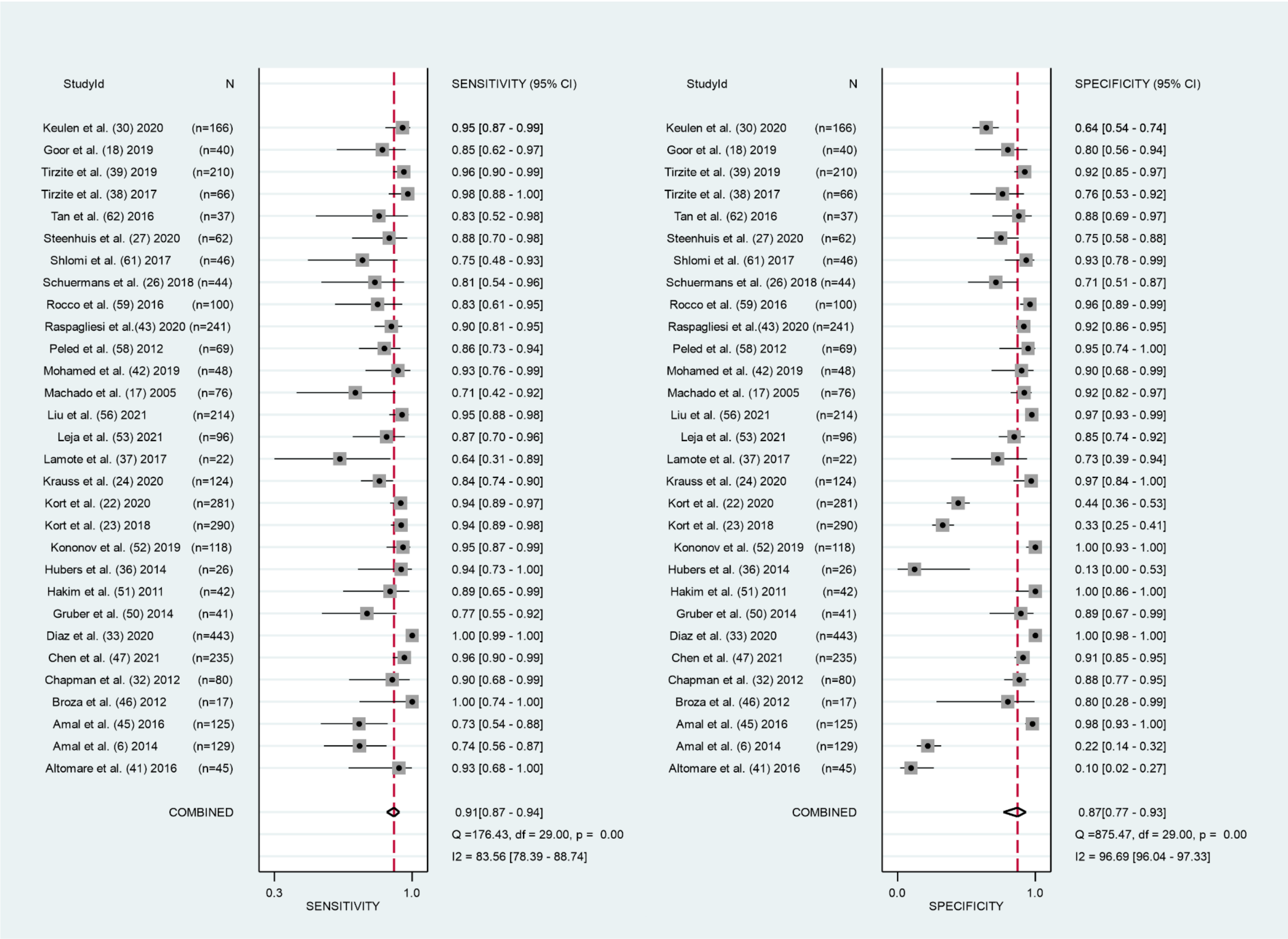

© 2022 Scheepers MHMC et al. *JAMA Network Open.*

eFigure 12: Pooled analysis of all studies after exclusion of studies with a high risk of bias on the Index Test domain of the QUADAS-2 tool

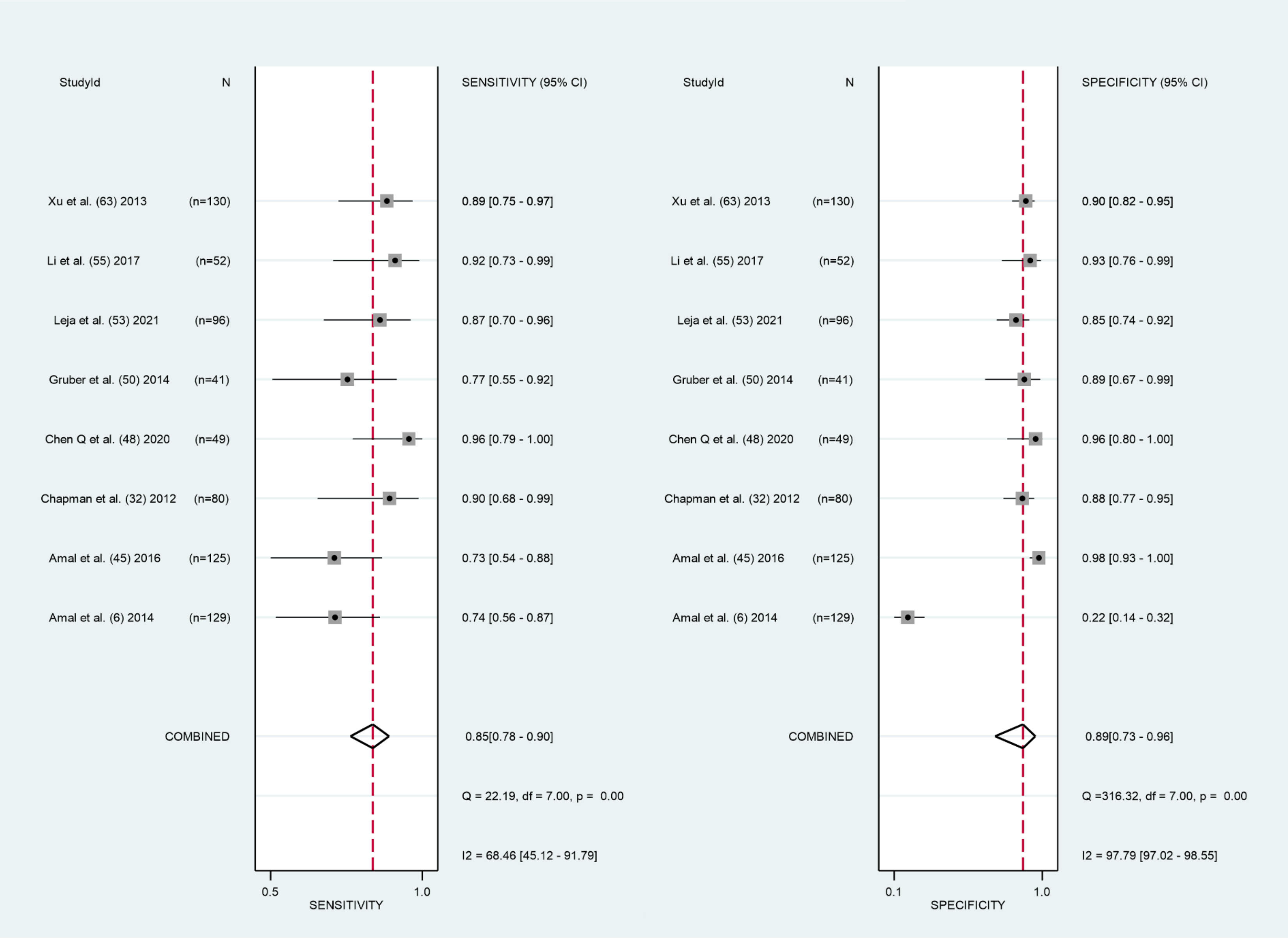

© 2022 Scheepers MHMC et al. *JAMA Network Open.*

eFigure 13: Pooled analysis of all studies after exclusion of studies with a high risk of bias on the Reference Standard domain of the QUADAS-2 tool

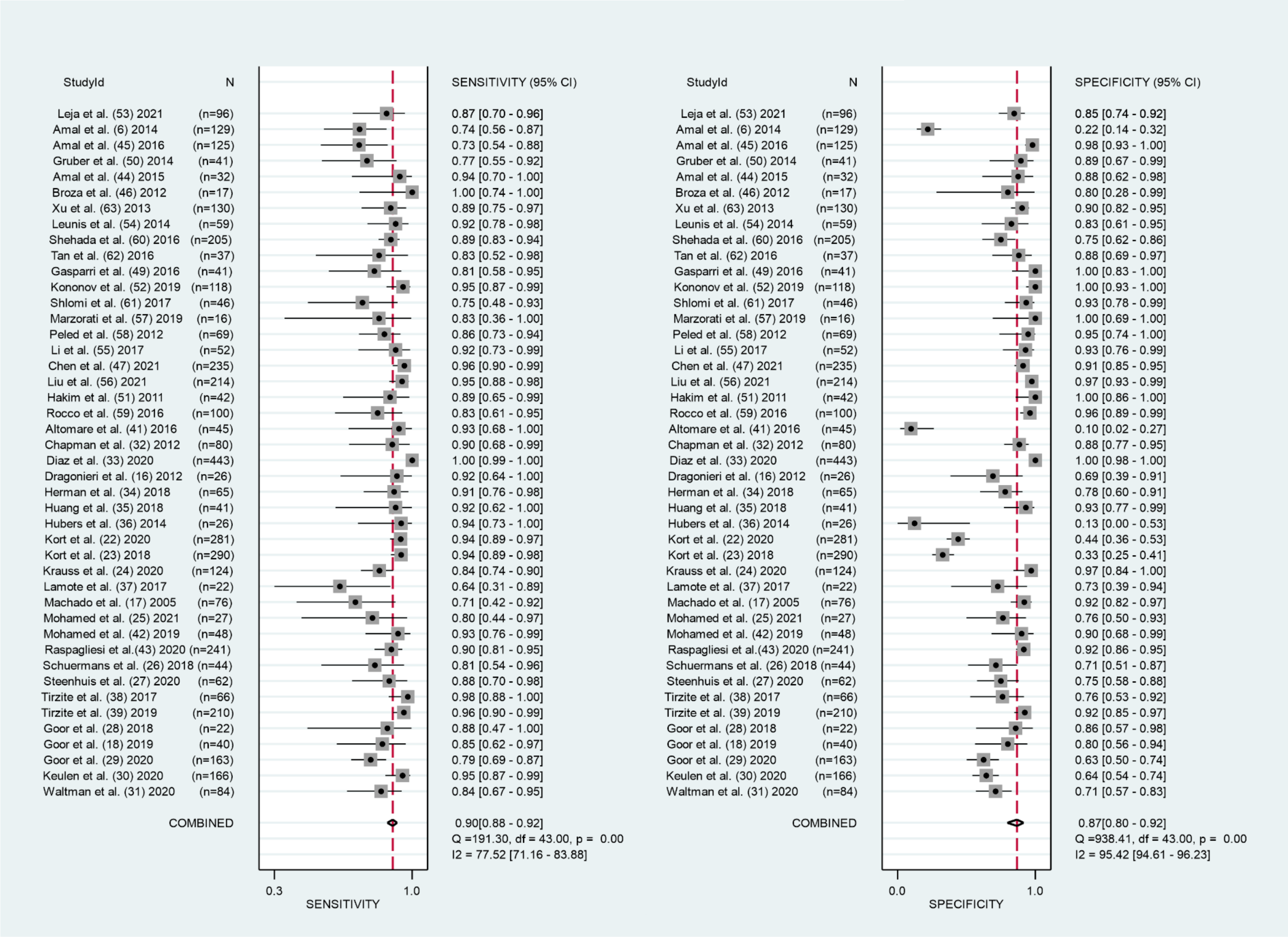

© 2022 Scheepers MHMC et al. *JAMA Network Open.*

eFigure 14: Pooled analysis of all studies after exclusion of studies with a high risk of bias on the Flow and Timing domain of the QUADAS-2 tool

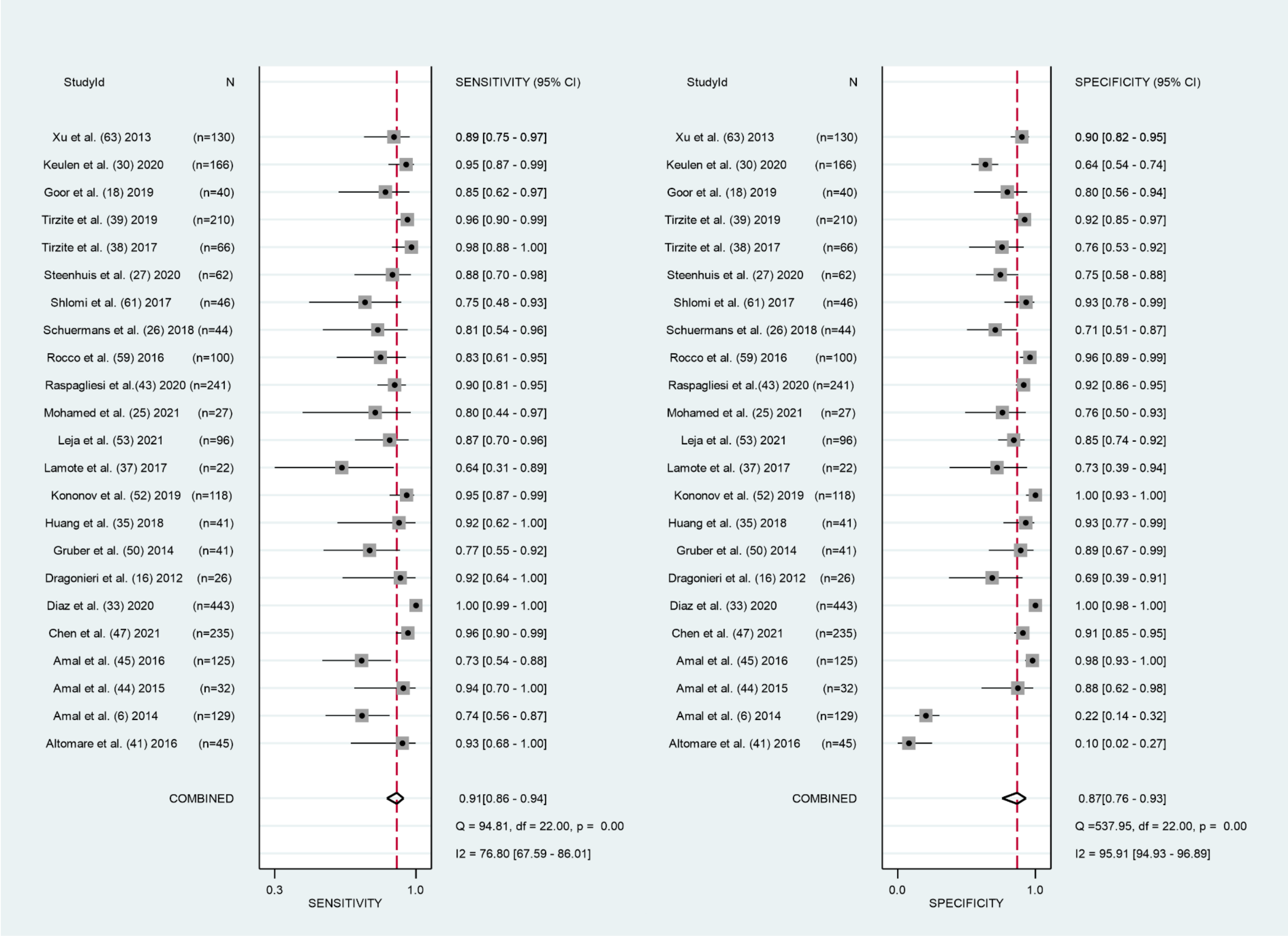

© 2022 Scheepers MHMC et al. *JAMA Network Open.*
